# Recent Advances in Wide‐Bandgap Perovskite Solar Cells

**DOI:** 10.1002/adma.202418622

**Published:** 2025-04-01

**Authors:** Jianjun Mei, Feng Yan

**Affiliations:** ^1^ Department of Applied Physics Research Center for Organic Electronics The Hong Kong Polytechnic University Hung Hom Kowloon Hong Kong SAR 999077 P. R. China

**Keywords:** applications, defects, photo‐instability, open‐circuit voltage deficits, solar cells, wide‐bandgap perovskites

## Abstract

Wide‐bandgap (WBG) perovskite solar cells (PSCs) have garnered considerable attention of late for their potential as semitransparent photovoltaics for building integration, top‐cells in tandem configurations, and indoor photovoltaics (IPVs) for Internet of Things (IoT) applications. However, recent investigations have unveiled that underlying defect‐mediated phase segregation, ion migration, lattice strain, and other factors can give rise to self‐accelerated degradation reactions and the contraction of quasi‐Fermi level splitting (QFLS) within devices. Extensive efforts have been undertaken to reduce defect densities in bulks, at surfaces, and across interfaces with charge transport layers (CTLs). This review provides a timely and comprehensive understanding of the intrinsic defect ecosystem in WBG perovskites, and mechanistically elucidates their impacts on device stability and open circuit voltage losses. Subsequently, recent advances in defect passivation strategies are cross‐sectionally overviewed, covering various components of devices. The applications of WBG PSCs in semitransparent devices, tandem applications, and IPVs are discussed. Finally, prospects and challenges are proposed, providing insights for future research and technological advancements.

## Graphical abstract

Ubiquitous defects predominately account for photo‐instability and open‐circuit voltage losses in wide‐bandgap perovskite solar cells (WBG PSCs). This review comprehensively presents the underlying impact mechanisms, summarizes the advanced optimization strategies across various functional layers and their interfaces to develop efficient and stable WBG PSCs, and evaluates their performance in semitransparent solar cells, tandem solar cells, and indoor photovoltaic applications.

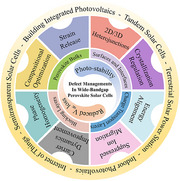

## 1 Introduction

In order to advance carbon neutrality initiatives, harnessing natural and ambient lights, as one of the renewable energies, to generate usable electricity stands out as a pivotal and effective strategy to combat the global climate disruptions caused by massive carbon dioxide emissions.^[^ ^1^, ^2^ ^]^ In a future visionary urban‐suburban paradigm,^[^ ^3^ ^]^ the expansive establishment of terrestrial photovoltaic power stations on the outskirts of cities and the seamless integration of solar cells into the architectural frameworks will emerge as the fundamental modes. Strategically deploying IPVs in human activity spaces is anticipated to sustainably energize wireless IoT smart devices and low‐power electronics.^[^ ^1^, ^4^ ^]^ These diverse array of application scenarios necessitates tailored considerations for photovoltaic material selections. For devices exposed to solar irradiance, photoactive materials are required to utilize the full solar spectrum as much as possible to maximize power conversion efficiency (PCE), and minimize energy loss from absorbed photons. Consequently, materials exhibiting suitable bandgaps of around 1.3 eV, according to the Shockley–Queisser (S‐Q) limit, are imperative for a single junction solar cell. Alternatively, a solution of stacking multiple sub‐cells with semiconductors of varying bandgaps, such as combining WBG materials that capture high‐energy photons with narrow‐bandgap (NBG) ones that harvest low‐energy photons, is more promising to optimize sunlight utilization.^[^ ^5^, ^6^, ^7^ ^]^ If there are additional requirements for the transparency and visual color aesthetics of devices (e.g. for bifacial photovoltaic cells and smart window applications), a trade‐off between these attributes and PCEs needs to be made.^[^ ^8^, ^9^, ^10^ ^]^ Unlike sunlight, absorber screening for IPV warrants meticulous consideration of two key factors: the compatibility between absorption spectra of materials and emission spectra (≈400–700 nm) of artificial light sources, and the high photo‐sensitivity to low levels of irradiance (two to three orders of magnitude lower than solar intensity).^[^ ^11^, ^12^ ^]^

WBG perovskites (*E*_g_ > 1.65 eV) can definitely engage in the applications mentioned above as prime candidates or crucial constituents due to their appealing properties, such as tunable bandgaps, high absorption coefficient, superior carrier dynamics, low exciton binding energy, and cost‐effective manufacturing.^[^ ^13^, ^14^, ^15^, ^16^ ^]^ Through facile halogen hybridization, the absorption onsets of lead halide WBG perovskites can be tuned within the visible light range, resulting in color transitions from deep reddish‐brown to light orange, corresponding to bandgap increments.^[^ ^9^ ^]^ Thanks to the high absorption coefficient property (over 10^4^ cm^−1^), an active layer in the thickness of a few hundred nanometers is proven adequate for capturing most of photons,^[^ ^8^, ^17^ ^]^ allowing a high degree of semi‐transparency. Despite the classical S‐Q theory suggests that WBG semiconductors perform sub‐optimally in single‐junction photovoltaic efficiency because of spectroscopic limits, their minimal hot‐carrier thermalization and increased open‐circuit voltages (*V*_oc_) with bandgaps render them suitable for tandem solar cells (TSCs).^[^ ^18^ ^]^ Theoretical proposals have suggested their utilization as top sub‐cells with NBG absorbers in mechanically or monolithically stacked configurations can deliver efficiencies of 46% for two‐junction setups and 50% for three‐junction devices, surpassing the 33.7% efficiency of single‐junction counterparts.^[^ ^7^, ^19^, ^20^, ^21^ ^]^ In fact, the state‐of‐the‐art achievements have validated it with the evidence of certified efficiencies of 34.6% PCE (for 1.69 eV perovskite/Si tandem cells) and 29.1% PCE (for ≈1.8 eV/1.25 eV all‐perovskite tandem cells), which outperforms those of single‐crystalline silicon cells (27.6%) and perovskite cells (26.7%) by a significant margin.^[^ ^22^, ^23^ ^]^ Additionally, extended studies grounded in the S‐Q theory have suggested that the optimum material bandgaps for IPVs should be around 1.9 eV, with maximum indoor efficiencies over 50–60% under white light‐emitting diodes (WLEDs) or fluorescent lamps (FLs).^[^ ^11^, ^24^, ^25^ ^]^ This suggests that there is ample room for improvement in effectiveness.

However, in practice, WBG perovskite devices suffer from the notorious photo‐instability and *V*_oc_ deficits, in which defects play a universal role. On the one hand, the incorporation of a large amount of bromine compounds (>20%) to broaden the bandgaps accelerates the nucleation and growth rate of the perovskite system, resulting in inhomogeneous chemical distribution and non‐uniform strain.^[^ ^26^, ^27^, ^28^, ^29^ ^]^ This process generates massive defects and reduces the migration activation energy of halide ions, making it prone to separate bromine‐rich and iodine‐rich phases upon photoexcitation. At the same time, ion migration may trigger a chemical reaction with electrodes and exacerbate the sensitivity of photoactive layers to external environmental factors (such as water and oxygen), ultimately leading to device failure.^[^ ^30^ ^]^ On the other hand, defects are widespread within the perovskite layers and CTLs, as well as at their interfaces, which increases non‐radiative carrier recombination and reduces the internal potential.^[^ ^31^, ^32^, ^33^ ^]^ The misalignment of interfacial energy levels raises the likelihood of defect‐assisted Shockley–Read–Hall (SRH) recombination.^[^ ^34^ ^]^ In recent years, important breakthroughs have been made in the field of WBG perovskite photovoltaics. Improvements in composition control,^[^ ^35^, ^36^ ^]^ additive engineering,^[^ ^37^, ^38^, ^39^ ^]^ solvent engineering,^[^ ^40^, ^41^ ^]^ and fabrication technology^[^ ^36^, ^42^ ^]^ have significantly gained high‐quality perovskite films with improved carrier lifetime and diffusion length. The development of new CTLs and interface passivation strategies ensure a better‐aligned level gradient and reduced defect state density, thereby increasing *V*_oc_.^[^ ^30^, ^43^, ^44^, ^45^ ^]^ In addition, innovative and diverse applications of barrier layers to improve device stability have been reported in some studies.^[^ ^46^, ^47^, ^48^, ^49^ ^]^ Overall, the performance of single‐junction WBG PSCs has ascended to new heights, with *V*_oc_ approaching or even exceeding 90% of S‐Q limit and operational stability achieving thousands of hours.

Given the large number of relevant research papers springing up, it is of great significance to sort out these findings and provide timely and comprehensive reviews. This review, from a defect‐centric perspective, begins by elucidating origins of photo‐instability and *V*_oc_ losses in bromine‐rich WBG PSCs. Subsequently, advanced defect management strategies are categorized and discussed, primarily concerning optimization methods for the perovskite layer, interface layer, charge transport layer, and blocking layer. Later, this review briefly outlines the remarkable progress of WBG perovskite cells in diverse applications, including semitransparent devices, TSCs, and IPVs. Finally, the prospects and challenges within this domain are proposed, with a wish to chart a course for future research endeavors and technological advancements.

## 2 Performance limitations of WBG PSCs

The limitations of high efficiencies and stable operation in Br‐rich WBG PSCs stem from two well‐established factors: “inferior photo‐instability” and “*V*_oc_ losses”. However, the exact mechanisms responsible for them are still subject to ongoing debates, despite the proposal of several possible aspects, including photo‐activated phase segregation,^[^ ^50^, ^51^, ^52^, ^53^ ^]^ ion migration,^[^ ^50^, ^54^, ^55^ ^]^ strain stress,^[^ ^56^, ^57^, ^58^ ^]^ and chemical inhomogeneity,^[^ ^29^, ^59^ ^]^ among others. Defects throughout the device appear to play a pivotal role in bridging these models. Nevertheless, diverse defect species, within perovskites, at both‐end surfaces and across interfaces with CTLs, add significant complexity to the situation. Consequently, this section aims to provide in‐depth insights into the underlying mechanisms of defect‐dominated instability and defect‐dependent *V*_oc_ losses in mixed halide WBG PSCs.

### 2.1 Defect‐Induced Instability

**Figure 1:**
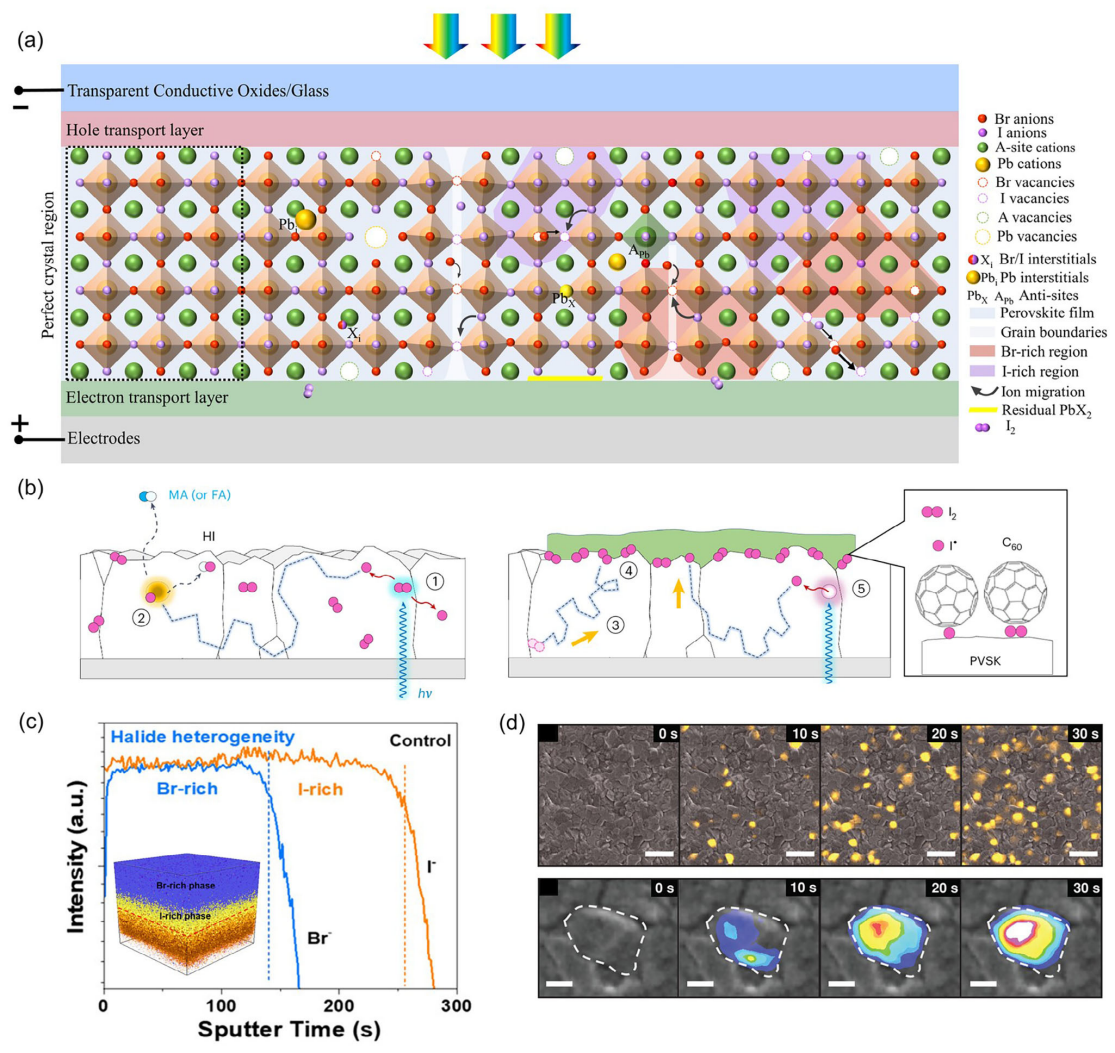
a) Illustrative diagram of a defective ecosystem within a typical bromine‐rich WBG PSC. b) Illustration of the self‐degradation of devices induced by the photolysis of iodine species: photolysis of I_2_ to iodine radicals ①; radical‐induced deprotonation of organic cations ②; long‐distance diffusion of I_2_ ③; capture of iodine species by C_60_ ④; photolysis of iodine radical from ideal perovskites ⑤. c) ToF‐SIMS I‐Br depth profile with the corresponding 3D rendered distribution overlay image inserted. d) Series of CL images at 2 µm and 200 nm scales captured at 10 s intervals during light soaking, where the color scale represents iodide‐rich CL intensity. b) Reproduced with permission.^[^ ^30^ ^]^ Copyright 2024, Springer Nature. c) Reproduced with permission.^[^ ^92^ ^]^ Copyright 2024, American Chemical Society. d) Reproduced with permission.^[^ ^79^ ^]^ Copyright 2017, American Chemical Society.

Currently, the prevailing WBG perovskite systems rely on Cs‐FA or all‐Cs bromine‐iodine hybrid compounds. Films fabricated through solution‐based or thermally evaporated techniques typically exhibit polycrystalline traits. Coupled with the soft ionic nature, perovskite films inherently host a multitude of intrinsic defects (**Figure** **1**a). Dimensionally, defects within perovskites can first be identified as zero‐dimensional defects, i.e., point defects, including various atom vacancies, and interstitial or full occupation of atoms in non‐local lattice positions (corresponding to interstitial and anti‐site defects, respectively). These defect types generally have increasing formation energies and are energetically distributed from shallow to deep in defect state levels.^[^ ^60^ ^]^ One‐dimensional defects are primarily characterized by linear dislocations at edge or screw positions, or their amalgamations. Two‐dimensional defects involve planar anomalies like defective surfaces or interfaces, grain boundaries, phase boundaries, facets, and twins. Both of them serve as reservoirs for various point defects. For instance, abundant Ruddlesden‐Popper anti‐phase boundaries were observed on an atomic scale in CsPbIBr_2_ via HAADF‐STEM, exhibiting uneven halide distribution, crystal displacement, element‐absent gaps, and peripheral lattice strain.^[^ ^61^ ^]^ Three‐dimensional bulk defects comprise precipitates (e.g., residual lead halides and metal lead clusters), heterogeneous phases, voids, and foreign impurities (e.g. dust), among others. In addition, other functional layers of solar cells are also accompanied by defects of varying concentrations and sorts. Examples include oxygen vacancy defects on the surface of oxide‐type transport layers, and undesired Ni^3+^ traps in NiO_x_.^[^ ^62^, ^63^, ^64^ ^]^

The formation of defects stems from a variety of mechanisms, with stoichiometric mismatch being a common cause. Bias may happen during manual weighing of raw materials, or during degradation of precursor solutions due to influences such as water, oxygen, light, and pH levels. Pertaining to the latter case, light‐sensitive iodine anions are readily oxidized to I_2_, depleting constituents in solutions.^[^ ^37^, ^65^, ^66^ ^]^ An abundance of uncoordinated iodine anions (I^−^) can generate negatively charged I_i_^−^ and neutral I_i_^0^ due to their lower formation energy.^[^ ^67^, ^68^ ^]^ These radicals trigger a reversible reaction with I_2_ vapor to transform I_3_^−^, a kind of I_i_^+^ point defect, during annealing. What's worse, I_3_^−^ has the potential to deprotonate FA^+^ cations, initiating volatile FA^0^ and I^0^ as well.^[^ ^37^, ^66^, ^68^, ^69^ ^]^ Post‐treatment procedures employing organic halide salts are reported to introduce interstitial iodine ions at surfaces unintentionally.^[^ ^70^ ^]^ Some organic CTLs, for example, C_60_, having non‐directional affinity for iodide or polyiodide species, would capture and transfer them to cause a deleterious reaction with metal electrodes and device self‐degradation (Figure 1b).^[^ ^30^ ^]^ Moisture‐induced phase transitions in CsPbX_3_ can give rise to X‐site vacancies and Pb^2+^ cations that preferentially accumulate along grain boundaries and surfaces.^[^ ^71^, ^72^ ^]^ Alloying elements in perovskites triggers non‐uniform nucleation dynamics (generally believed to be n(Cs^+^) > n(FA^+^), n(Br^−^) > n(I^−^)), resulting in uncontrolled crystal growth, lattice mismatches, and component aggregation or delamination (Figure 1c).^[^ ^29^, ^73^ ^]^ It further gives rise to plenty of defects and heterogeneous phase boundaries. Incorporating lead‐containing additives brings in PbX_2_ impurities,^[^ ^74^ ^]^ which is also ascribed to the potential byproducts of the PbX_2_‐DMSO intermediate phase.^[^ ^75^, ^76^ ^]^ Upon exposure to high‐energy photons, PbX_2_ decomposes into metallic Pb^0^ and X_2_ molecules.^[^ ^77^ ^]^ Pb^0^ obstructs the crystallization process and sparks the generation of deep‐level traps. Moreover, the discrepancy in thermal expansion coefficient between the substrate materials and the perovskite lattice can form the out‐of‐plane tensile strain.^[^ ^26^ ^]^ Such strain, in turn, induces more ion vacancies by the piezoelectric effect and activates a vicious cycle of strain aggravation.^[^ ^28^, ^78^ ^]^

The defect‐induced photo‐instability in WBG PSCs is evident. Hoke et al. were the first to document the reversible light‐activated spectral red‐shift in mixed halide perovskites, attributing it to the segregation of I‐rich and Br‐rich phases.^[^ ^53^ ^]^ Ginsberg et al. utilized the cathode luminescence (CL) imaging technique to observe the temporal evolution of phase segregation at a nano‐optical scale (Figure 1d).^[^ ^79^ ^]^ In light soaking, I‐rich crystal domains preferentially germinated rapidly at grain boundaries, edges between different planes, and surfaces, followed by gradual expansion. Several mainstream mechanism models have been proposed to explain the underling origins of phase segregation. The thermodynamic phase diagram of the MAPb(I_1‐x_Br_x_)_3_ system was first presented by Brivio et al., revealing that highly alloyed halides (0.3 <  *x*  <  0.6) are thermodynamically metastable at room temperature, with intense light exposure overcoming the kinetic barrier to exacerbate it.^[^ ^80^ ^]^ Subsequently, Draguta et al. suggested that the valence edge variances between different phases were the energetic impetus that counteracts the thermodynamic entropy preference toward mixed phases.^[^ ^81^ ^]^ Polaron‐induced strain model assumes that photogenerated free charges would either be bound by deep‐level defects or captured at the heterointerface of the perovskite and CTLs,^[^ ^82^, ^83^ ^]^ leading to localized charge accumulation and lattice distortion through electron‐phonon coupling.^[^ ^53^, ^79^, ^84^ ^]^ The unstable lattice structure randomly spawns nano‐scale I‐rich clusters capable of capturing polaritons. These polaritons, in turn, render more iodide ions to migrate and aggregate, rooting for phase separation. The electric field‐driven model proposes that the localized electric field, generated by carrier gradients under intense illumination^[^ ^85^ ^]^ or by defect‐confined photocarriers,^[^ ^86^, ^87^ ^]^ lies at the root of halide segregation. Electrostatic interactions with vacancy defects prompt the migration of halide ions. Due to the lower migratory activation energy of iodide ions compared to bromine,^[^ ^88^, ^89^, ^90^ ^]^ iodide transport (in the form of Schottky or Frenkel defect motion^[^ ^78^ ^]^) predominates, leading to a net halide segregation.

Noted that, in real‐world outdoor and testing conditions, prolonged exposure to intense light can generate accumulated heat. Elevated temperatures and external voltage bias can make the above situations worse.^[^ ^30^, ^91^ ^]^ Although photo‐induced phase segregation is reversible, the ion migration and photolysis products (e.g., I_2_) occurring during this process may degrade device due to reactions with metal electrodes and permanent outflows of halides. Consequently, defect passivation becomes a primary consideration in WBG PSCs, attainable through strategies such as chemical homogenization, strain release, and inhibition of phase segregation and ion migration, among others.

### 2.2 Defect‐Dependent *V*_oc_ Losses

The QFLS theory has been extensively evaluated in the analytical interpretation of *V*_oc_ loss in the photovoltaic discipline.^[^ ^33^, ^93^, ^94^, ^95^ ^]^ QFLS delineates the divergence between the fermi levels of electrons and holes, stemming from the non‐equilibrium quantities of photogenerated free carriers in bulks under illumination. It provides a microscopic insight into the internal electrochemical potential. Once in direct contact with an external voltmeter, this implied voltage can be discernible, thereby deriving *V*_oc_. Under ideal circumstances of flawless contact, QFLS and *V*_oc_ are deemed as interchangeable physical quantities. At the energetic level, their correlation can be explicated through the free charge carrier densities (*n*_e_ for electrons and *n*_h_ for holes), according to the classic semiconductor theory:^[^ ^96^, ^97^ ^]^

(1) $$q\cdot V_{\mathrm{oc}}\approx QFLS=kT\ln\;\left(\frac{n_{e}n_{h}}{n_{i}^{2}}\right)=E_{g}\;-kT\ln\left(\frac{N_{c}N_{v}}{n_{e}n_{h}}\right)$$

where *q* is the elementary charge, *k* is the Boltzmann constant, *T* is the temperature in Kelvin, *n*_i_ is the intrinsic equilibrium carrier density, *E*_g_ is the bandgap energy of photoactive materials, *N*_c_ and *N*_v_ signify the effective densities of occupied states in the conduction and valance bands, respectively. This equation indicates that, at finite temperature, the more charge carrier density there is, the higher *V*_oc_ and QFLS to achieve.

**Figure 2:**
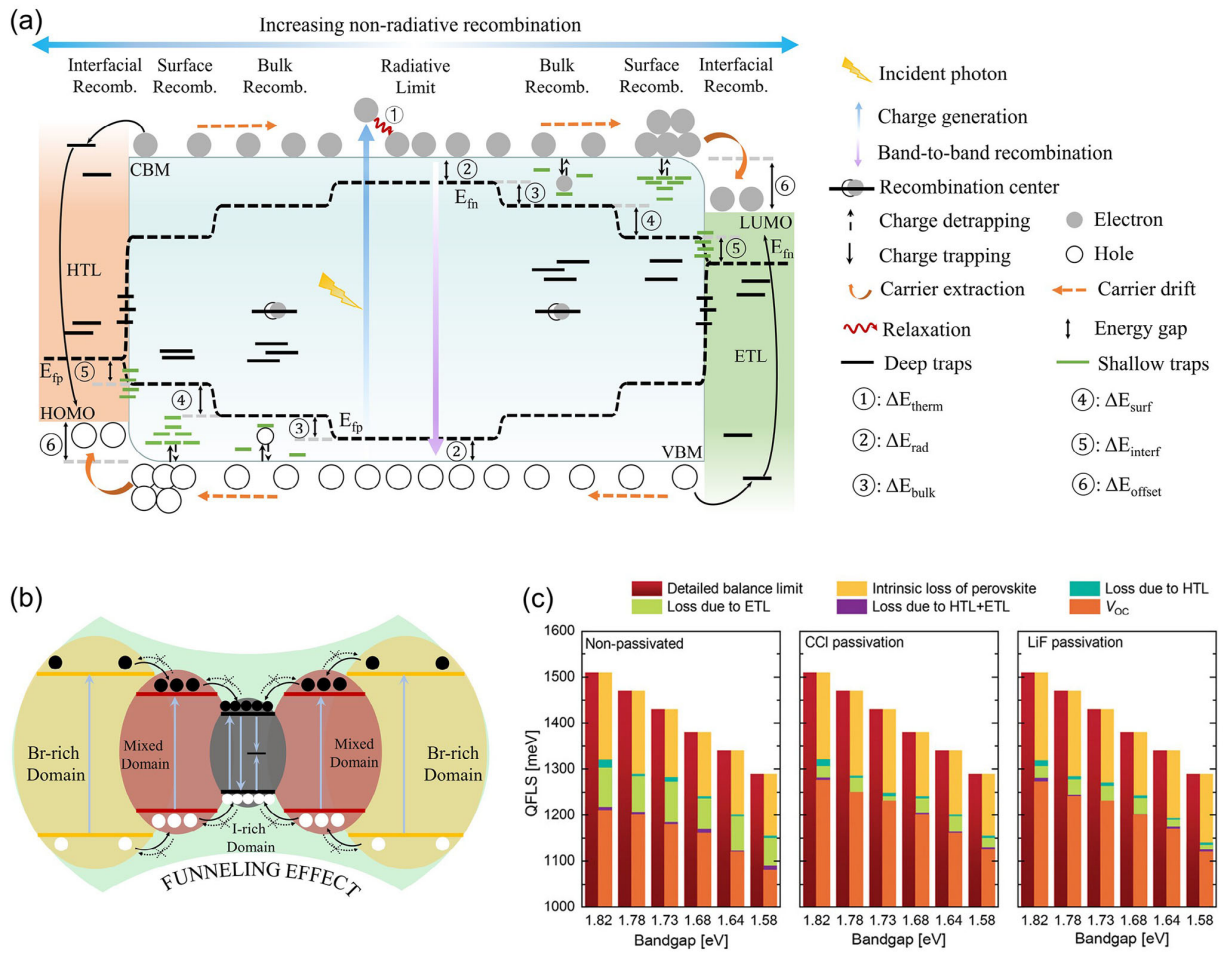
a) Schematic diagram of charge transfer and recombination pathways in PSCs, alongside the affected QFLS. b) Photogenerated carriers trapped in I‐rich domains by funneling effect under phase segregation. c) Comparisons of QFLS loss contributions from neat photoactive films and various cell stacks with different bandgap perovskites and surface passivation treatments (CCl for chemical passivation and LiF for physical passivation). c) Reproduced with permission.^[^ ^33^ ^]^ Copyright 2024, Wiley‐VCH GmbH.

In realistic, the population of free carriers in PSCs will inevitably encounter initial constraints dictated by thermal equilibrium and radiative recombination, followed by succumbing to the sway of non‐radiative recombination instigated by ubiquitous defects throughout the device. **Figure** **2**a paints charge generation, transfer and recombination pathways in a prototypical PSC. In the primary phase, electrons transit to the conduction band only by absorbing photons carrying energies surpassing the *E*_g_ threshold of materials, consequently leaving holes behind in the valence band. If the photoexcited electrons reach high excited states, they ultimately settle into the lowest energy state within the conduction band via releasing excessive energy in the manner of thermal radiation (① in Figure 2a). This thermal energy, in conjunction with other heat (e.g. generated by unabsorbed photons), imbues solar cells with a specific temperature, upholding the internal carrier concentration in a state of thermodynamic equilibrium. At an open‐circuit condition, the net current converges to zero, implying a cancellation between the photocurrent (*I*_ph_) and recombination current (*I*_R_), or a parity between the generation rate (*G*) and recombination rate (*R*) of carriers. Revisiting the detailed balance proposed by S‐Q theory,^[^ ^18^ ^]^ if a singular recombination pathway govern by band‐to‐band recombination (i.e., 100% radiative recombination, *G *= *R *= *n*_e_ *n*_h_), the *V*_oc_ can be extrapolated from the Shockley diode equation:^[^ ^98^ ^]^

(2) $$V_{\mathrm{oc}}^{\mathrm{SQ}}=\frac{kT}{q}\;\;\ln\left(\frac{J_{\mathrm{ph}}}{J_{0}}+1\right)=\frac{kT}{q}\;\ln\left(\frac{\int_{E_{g}}^{\infty }q\emptyset_{\mathrm{AM}1.5G}\left(E\right)dE}{\int_{E_{g}}^{\infty }q\emptyset_{\mathrm{BB}}\left(E\right)dE}+1\right)$$

where photocurrent density (*J*_ph_) and dark saturation current density (*J*_0_) can be integrated over the energy scale from the AM1.5G irradiance spectrum (*Φ*_AM1.5G_) and the emitting spectrum (*Φ*_BB_) of a black‐body radiation, respectively. This formula reciprocally links to Equation (1), bridging “thermodynamic radiative view” and “semiconductor picture,”^[^ ^99^ ^]^ predicting the theoretical maximum achievement of $V_{\mathrm{oc}}^{\mathrm{SQ}}$ and QFLS. Based on this, $V_{\mathrm{oc}}^{\mathrm{SQ}}$ scales with *E*_g_, for example, 1.418 V $V_{\mathrm{oc}}^{\mathrm{SQ}}$ for 1.7 eV‐perovskites, 1.512 V $V_{\mathrm{oc}}^{\mathrm{SQ}}$ for 1.8 eV‐perovskites and 1.605 V $V_{\mathrm{oc}}^{\mathrm{SQ}}$ for 1.9 eV‐perovskites.

Considering extra thermal radiation in a solar cell in dark, an energy loss (② in Figure 2a) may happen. Since the external quantum efficiency (*EQE*_PV_) of devices extends to the sub‐bandgap region (below *E*_g_), the black‐body radiation is thereby augmented.^[^ ^100^ ^]^ It can marginally shrink the QFLS domain further. Equation (2) can be rewritten as:

(3) $$\begin{array}{ccc} V_{\mathrm{oc}}^{\mathrm{rad}} & = & \frac{kT}{q}\;\;\ln\left(\frac{J_{\mathrm{ph}}}{J_{0,rad}}+1\right)\\ & = & \frac{kT}{q}\;\ln\left(\frac{\int_{0}^{\infty }qEQE_{\mathrm{PV}}\emptyset_{\mathrm{AM}1.5G}\left(E\right)dE}{\int_{0}^{\infty }qEQE_{\mathrm{PV}}\emptyset_{\mathrm{BB}}\left(E\right)dE}+1\right) \end{array}$$

where *J*_0,rad_ represents the saturated dark current density under the circumstance of radiative recombination. From the carrier perspective, *R* now becomes proportional to *n*_e_ *n*_h_ with a second‐order recombination rate constant (*β*_r_).^[^ ^101^ ^]^ Consequently, Equation (1) can be refined as:

(4) $$q\cdot V_{\mathrm{oc}}^{\mathrm{rad}}\approx \;QFLS_{\mathrm{rad}}=E_{g}\;-kT\ln\left(\frac{N_{c}N_{v}\beta_{r}}{G}\right)$$

Fortunately, for perovskites characterized by sharp absorption edges, this yields negligible voltage losses,^[^ ^102^ ^]^ typically a few tens of millivolts for solar cells fashioned from WBG perovskites.^[^ ^100^, ^103^ ^]^

Beyond radiative recombination, other recombination pathways are uniformly classified as non‐radiative recombination, in which defect recombination (also recognized as trap‐assisted recombination or SRH recombination) takes precedence. Defects, arising from foreign impurities and internal crystal imperfections (such as point defects, dislocations, grain boundaries, etc.), can either act as shallow traps that capture and de‐capture carriers, or serve as deep‐level traps that annihilate electron‐hole pairs. The evaluation criteria for this are associated to the defect energy level position, defect density, and capture cross‐sectional area, and their charge selectivity.^[^ ^104^ ^]^ In particular, deep traps are generally acknowledged as strong recombination centers, rather than shallow traps. Within perovskite bulks, the SHR recombination rate (*R*_Bulk_) is determined by the non‐radiative lifetimes of electrons (τ_e_) and holes (τ_h_):^[^ ^101^ ^]^

(5) $$R_{Bulk}=\frac{n_{e}n_{h}}{\tau_{e}n_{e}+\tau_{h}n_{h}}\;$$

Conversely, at perovskite surfaces, the recombination rate (*R*_surf_) is characterized by the recombination of excess minority carriers (Δ*n* for electrons, and Δ*h* for holes), which is mathematically related to their surface recombination velocity ($\upsilon_{e}\;or\;\upsilon_{h}$):^[^ ^105^ ^]^

(6) $$R_{\mathrm{surf}}=\upsilon_{e}\;\Delta n\;or=\upsilon_{h}\;\Delta h$$

Upon incorporating CTLs to sandwich the perovskite layer, the recombination dynamics (*R*_int_) becomes more complexed.^[^ ^106^ ^]^ Take perovskite/CTL interface as an example, *R* is articulated as:

(7) $$R_{\mathrm{int}}=\frac{n_{e,\mathrm{ETL}}n_{h,\mathrm{PVSK}}-n_{i,\mathrm{int}}^{2}}{n_{e,\mathrm{ETL}}/\upsilon_{h}+n_{h,\mathrm{PVSK}}/\upsilon_{e}}$$

where $n_{i,\mathrm{int}}^{2}$ is the intrinsic equilibrium carrier density at interface, which is proportional to $e^{-E_{int}/kT}$, with *E_int_* representing the energy offset of conduction‐band edges of the two layers.

SRH recombination occurring in bulks, at surfaces and interfaces gives rise to the energy losses corresponding to ③ – ⑥ in Figure 2a, respectively, significantly influencing the splitting of quasi‐fermi levels. Joint with these factors, a more realistic *V*_oc_ can be expressed as:

(8) $$V_{oc}=\frac{kT}{q}\;\ln\left(\frac{J_{ph}}{J_{0,rad}+J_{0,\mathrm{non}-\mathrm{rad}}}+1\right)$$

where *J*_0,non‐rad_ is the total dark saturation dark current density produced by all non‐radiative recombination pathways.

To quantify non‐radiative recombination in voltage loss ($\Delta V_{\mathrm{oc}}^{\mathrm{non}-\mathrm{rad}}$), two popular characterization methodologies, absolute photoluminescence and electroluminescence, can be accessible. Their quantum yields have a relationship with $\Delta V_{\mathrm{oc}}^{\mathrm{non}-\mathrm{rad}}$ as follows:

(9) $$\Delta \;V_{\mathrm{oc}}^{\mathrm{non}-\mathrm{rad}}=\frac{kT}{q}\;\;\ln\left(PLQY^{-1}\right)=\frac{kT}{q}\;\ln\left(EQE_{\mathrm{EL}}^{-1}\right)$$

where PLQY and EQE_EL_ refer to photoluminescence quantum yield and electroluminescence external quantum efficiency, respectively.

In WBG perovskite materials, identified deep‐level traps include under‐coordinated halides (X^−^) or Pb^2+^, Pb clusters, PbI_2_, and Pb‐X anti‐site defects (PbX_3_^−^).^[^ ^83^, ^107^ ^]^ Shallow‐level A‐ or X‐site vacancies increase the concentration of recombination centers within devices by sparking a chain of optical reactions or degraded responses, instead of directly participating in the elimination of photogenerated carriers. Distorted grains or dislocations disrupt the systematic energy, intensifying non‐radiative recombination.^[^ ^37^ ^]^ Additionally, in the case of phase segregation, both electrons and holes (especially holes) will be channeled into funnel‐shaped snares via energy gradients, leading to internal annihilation (Figure 2b). Therefore, I‐rich phases could also be viewed as deep‐level traps. It is noteworthy that the contribution of photo‐induced phase segregation to *V*_oc_ deficit is subsidiary, time‐dependent and not uniformly quantifiable. Prior to the phenomenon, SRH recombination mediated by the intrinsic defects is the main channel for the initial voltage decline. A minor part of this might be attributed to transport barrier within different phase domains due to inhomogeneous distribution of halide anions.^[^ ^108^ ^]^ Afterward, the phase transition is induced by ion migration, accompanied by the generation of ion vacancies, I‐rich regions at grain boundaries, and Pb‐related deep defects. It results in an additional voltage drop, which is determined by the duration of light soaking, the speed and extent of phase separation, and the resulting density of newly introduced defect states.^[^ ^109^, ^110^, ^111^, ^112^ ^]^ Certain reports have quantitatively assessed this loss to be ≈100 mV only.^[^ ^110^, ^111^ ^]^

Remmerswaal et al. observed that non‐radiative recombination at perovskite/electron transport layer (ETL) interface resulted in increased voltage losses with widening bandgaps, second to intrinsic losses (Figure 2c).^[^ ^33^ ^]^ Whether through chemical passivation to vacancies or physical isolation by interlayers to prevent direct interfacial contact, significant enhancements in *V*_oc_ could be achieved. Stolterfoht et al. stressed the importance of interface energy level alignment and highlighted that unfavorable pairs might exponentially rise non‐radiative recombination.^[^ ^34^ ^]^ Equation (7) echoes this viewpoint. Indeed, the presence of a large interface energy barrier hinders the efflux of majority carriers, provoking the accumulation of charges at the interface and instigating an asymmetry in carrier concentration on both sides.^[^ ^93^, ^113^ ^]^ The accumulated charge region draws opposite charges closer via electrostatic attraction, accelerating the interfacial recombination rate. Furthermore, Caprioglio et al. advocated for considering the charge extraction selectivity of CTLs, as carriers could be transferred through special traps within CTLs to recombine with counter charges, leading to voltage saturation (see Figure 2a).^[^ ^95^ ^]^

To sum up, the total *V*_oc_ deficit consists of three major parts mentioned above:

(10) $$\begin{array}{c} \Delta \;V_{\mathrm{oc}}=\frac{E_{g}}{q}\;-V_{\mathrm{oc}}=\left(\frac{E_{g}}{q}-V_{\mathrm{oc}}^{\mathrm{SQ}}\right)+\left(V_{\mathrm{oc}}^{\mathrm{SQ}}-V_{\mathrm{oc}}^{\mathrm{rad}}\right)+\\ \left(V_{\mathrm{oc}}^{\mathrm{rad}}-V_{\mathrm{oc}}\right)=\Delta V_{1}+\Delta V_{2}+\Delta V_{3} \end{array}$$

where Δ*V*_1_, Δ*V*_2_, and Δ*V*_3_ are referred to thermodynamic loss, radiative recombination loss and non‐radiative recombination loss, respectively. More efforts should be devoted to mitigating the defect density throughout the device in an effort to bring *V*_oc_ close to its theoretical limit.

## 3 Defect Management in PSCs

Defect passivation engineering stands as a fundamental yet critical pillar in the endeavor to ameliorate WBG PSCs’ efficiency and stability. This intricate process entails the meticulous optimization of intrinsic perovskite bulks, CTLs, interfacial regions, and blocking layers, considering the ubiquitous defects or the adverse reactions caused by defects throughout the whole device configuration. Consequently, in the forthcoming discourse, we will delve into a range of efficacious strategies reported recently, spanning from various tiers of the holistic device architecture to manufacturing technologies, which bring current status to unprecedented heights (**Table** **1**).

**Table 1:** Summary of photovoltaic performance and operational stability of state‐of‐the‐art single‐junction WBG PSCs.

*E*_g_ [eV]	Configuration	*V*_oc_ [V]	*J*_sc_ [mA cm^−2^]	FF [%]	PCE [%]	Stability	Refs.
1.65	ITO/4PADCB/FA_0.8_Cs_0.15_MA_0.05_PbI_2.4_Br_0.6_ (by **DMPU:DMF:DMSO**)/PADI_2_/C_60_/BCP/Cu	1.256	21	83	21.9	*T*_90_ = 1000 h@unencap., AM1.5G, 15–25% RH, air; MPP, *T*_94_ = 200 h@encap., 1‐sun, 20 °C, 20–35% RH	[37]
1.65	ITO/**Cu:NiO_x_**/Me‐4PACz/Cs_0.25_FA_0.75_Pb(I_0.85_Br_0.15_)_3_/C_60_/SnO_x_/Ag	1.22	22.16	83.1	22.47	n.a.	[63]
1.65	ITO/SnO_x_/FA_0.83_Cs_0.17_Pb(I_0.8_Br_0.2_)_3_:**PbI_2_**/PEAI/Spiro‐OMeTAD/MoO_x_/Ag	1.266	20.78	77.54	20.41	n.a.	[74]
1.65	ITO/Me4‐PACz/Al_2_O_3_/Cs_0.25_FA_0.75_Pb(I_0.85_Br_0.15_)_3_:**DMAFo**/PADAI/C_60_/SnO_x_/Ag	1.206	22.42	81.55	22.05	MPP, *T*_100+_ = 1000 h@encap., 1‐sun LED, 40 °C, air; MPP, *T*_100+_ = 1000 h@unencap., 1‐sun LED, 40 °C, N_2_; *T*_100_ = 1200 h@85 °C, N_2_	[37]
1.66	ITO/**TZO(O)**/SnO_2_/FA_0.83_Cs_0.17_Pb(I_0.8_Br_0.2_)_3_/MABr/Spiro‐OMeTAD/Au	1.241	20.89	83.89	21.62	*T*_89.47_ = 1000 h@unencap., 25 °C, N_2_	[266]
1.66	ITO/NiO_x_/Me‐4PACz/Cs_0.2_FA_0.8_Pb(I_0.8_Br_0.2_)_3_/**GuSCN**/C_60_/SnO_x_/Ag	1.2	21.26	82.04	20.92	*T*_95.4_ = 3384 h@unencap., RT, N_2_; *T*_86.6_ = 2112 h@unencap., RT, 12% RH, air	[273]
1.66	ITO/MeO‐2PACz/Cs_0.05_FA_0.8_MA_0.15_Pb(I_0.8_Br_0.2_)_3_/**AlO_x_**/C_60_/SnO_2_/Ag	1.181	22.59	81.72	21.8	*T*_97_ = 3500 h@AM1.5G, N_2_; *T*_90_ = 1300 h@RT, 35% RH, air; *T*_87 _= 1560 h@unencap., 100 mW cm^−2^ LED, N_2_; *T*_78_ = 672 h@unencap., 100 mW cm^−2^ LED, 65 °C, N_2_	[46]
1.66	FTO/SnO_x_/FA_1‐x_Cs_x_Pb(I_1‐y_Br_y_)_3_:**FAAc**/Spiro‐OMeTAD/Au	1.154	20.95	81.3	19.49	*T*_90_ = 1500 h@unencap., 25 °C, 50–60% RH, air; *T*_80_ = 600 h@unencap., 60 °C, N_2_	[274]
1.67	FTO/c‐TiO_2_/mp‐TiO_2_/Cs_0.05_FA_0.72_MA_0.23_Pb(I_0.77_Br_0.23_)_3_/**FABr:2PACz**/Spiro‐OMeTAD/Au	1.198	20.5	81	19.8	*T*_95_ = 2800 h@unencap., AM1.5G, RT, 40% RH, air	[227]
1.67	ITO/Me‐4PACz/Cs_0.17_FA_0.83_Pb(I_0.77_Br_0.23_)_3_/**AEAPTMS**/PCBM/BCP/	1.26	19.5	84	20.6	n.a.	[43]
1.67	ITO/MeO‐2PACz/Cs_0.25_FA_0.75_Pb(I_0.8_Br_0.2_)_3_:**DBT**/C_60_/BCP/Cu	1.247	20.72	85.7	22.12	*T*_90_ = 500 h@unencap., 100 mW cm^−2^ LED, N_2_	[163]
1.67	ITO/NiO_x_/Me‐4PACz/Cs_0.25_FA_0.75_Pb(I_0.75_Br_0.25_)_3_:**[MMP][SCN]**/C**_60_**/BCP/Ag	1.14	20.98	84.12	20.24	MPP, *T*_91.5_ = 1000 h@unencap., 1sun LED, air; *T*_82.2_ = 700 h@unencap., 85 °C, N_2_; *T*_90.2_ = 600 h@unencap., 80–85% RH, air	[149]
1.67	ITO/Me‐4PACz/Cs_0.05_MA_0.22_FA_0.73_Pb(I_0.77_Br_0.23_)_3_/**SEBr**/LiF/C_60_/BCP/Ag	1.28	20.37	84.46	22.47	*T*_80_ = 1000 h@unencap., 20–30% RH, 25 °C, air; *T*_90_ = 1000 h@1‐sun, N_2_	[257]
1.67	ITO/PTAA/OAI/DMA_0.1_Cs_0.25_MA_0.05_FA_0.6_Pb(I_0.9_Br_0.1_)_3_/**AET‐HCl**/C_60_/BCP/Ag	1.25	20.27	82.98	21.02	MPP, T_80_ = 500 h@1‐sun LED, 60 °C, N_2_	[275]
1.67	ITO/NiO_x_/Me‐4PACz/FA_0.7_Cs_0.25_MA_0.05_Pb(I_0.8_Br_0.2_)_3_:ODADI/PDADI/C_60_/BCP/Cu	1.23	19.96	84.4	22.06	MPP, T_100_ = 500 h@unencap., 1‐sun LED, 40 °C, air	[276]
1.67	tFTO/SnO_x_/Cs_0.17_FA_0.83_Pb(I_0.8_Br_0.2_)_3_:MACl/**BABr**/Spiro‐OMeTAD/Au	1.225	20.57	81.2	20.46	n.a.	[277]
1.67	ITO/Me‐4PACz/Cs_0.05_FA_0.80_MA_0.15_Pb(I_0.75_Br_0.25_)_3_/**surface polishing by AlO_x_ NPs**/PI/C_60_/BCP/Ag	1.29	22.32	85	24.48	MPP, T_80_ = 1505 h@100 mW cm^−2^ LED, N_2_	[189]
1.68	FTO/NiO_x_/VNPB/Cs_0.3_DMA_0.2_MA_0.5_PbI_3_ (by **anti‐solvent‐free**)/PCBM/BCP/Ag	1.193	21.7	82.3	21.3	MPP, *T*_88_ = 1000 h@encap., 100 mW cm^−2^ WLED, 25 °C, 30–40% RH, air *T*_92.6_ = 1900 h@unencap., 25 °C, 40% RH, air; *T*_91.2_ = 250 h@unencap., 65 °C, N_2_	[41]
1.68	ITO/**AlO_x_**/SnO_2_/Cs_0.05_FA_0.8_MA_0.15_Pb(I_0.75_Br_0.25_)_3_/PEAI/Spiro‐OMeTAD/Au	1.26	21.5	79.4	21.5	n.a.	[270]
1.68	a‐NbO_x_/**C_60_‐SAM/F‐PEABr**/Cs_0.05_FA_0.8_MA_0.15_Pb(I_0.755_Br_0.255_)_3_/PEABr:ImBr/Spiro‐TTB:F6‐TCNNQ/Ag	1.21	21.8	77.3	20.4	n.a.	[230]
1.68	ITO/PTAA/FA_0.8_Cs_0.2_Pb(I_0.8_Br_0.2_)_3_:**RbI:RbBr:MACl**/PEAI/PCBM/BCP/Ag	1.22	21.48	82.88	21.72	*T*_90_ = 1500 h@unencap., 1‐sun, N_2;_ *T*_86_ = 1500 h@unencap., 25 °C, 30–40% RH, air	[129]
1.68	ITO/4PADCB/FA_0.8_Cs_0.2_Pb(I_0.8_Br_0.2_)_3_ (by **Close Space Annealing** with F‐PEACl:IPA)/F‐PEACl/C_60_/SnO_x_/ITO	1.248	20.77	82.1	21.28	*T*_95_ = 1008 h@AM1.5G, N_2_	[185]
1.68	ITO/NiO_x_/Me‐4PACz/Cs_0.05_FA_0.8_MA_0.15_PbI_2.25_Br_0.75_/**APA‐3**/C_60_/BCP/Ag	1.216	22.18	82.87	22.35	MPP, *T*_90_ = 985 h@100 mW cm^−2^ LED; *T*_80_ = 500 h@85 °C, N_2_	[260]
1.68	FTO/poly‐TPD/Al_2_O_3_ NPs/FA_0.75_Cs_0.25_Pb(I_0.8_Br_0.2_)_3_:**BnAm**/PCBM/BCP/Cr/Au	1.22	21.62	80	21.1	*T*_80_ = 2460 h@encap., 65 °C	[168]
1.68	ITO/NiO_x_/Poly‐TPD/Cs_0.22_FA_0.78_Pb(I_0.85_Br_0.15_)_3_:**TFABI**/PCBM/BCP/Au	1.17	21.78	84.33	21.54	*T*_95_ = 1400 h@unencap., 25 °C, N_2_; *T*_92_ = 60 h@(60 mW cm^−2^) 365 nm UV, 25–30% RH, 25 °C	[278]
1.68	ITO/**MeO‐PhPACz:Me‐4PACz**/Cs_0.05_FA_0.8_MA_0.15_Pb(I_0.75_Br_0.25_)_3_/C_60_/BCP/Ag	1.236	21.12	86.67	22.63	MPP, *T*_80_ = 800 h@unencap., 1‐sun, RT, N_2_; *T*_90_ = 900 h@encap., 85% RH, 85 °C, day/night cycle	[225]
1.68	ITO/2PACz/Cs_0.05_FA_0.8_MA_0.15_Pb(I_0.75_Br_0.25_)_3_/CcPF_6_/C_60_/SnO_x_/Ag	1.28	19.87	81.71	20.78	*T*_93.24_ = 1000 h@unencap., 5% RH, 20 °C, air	[279]
1.68	ITO/MeO‐2PACz/FA_0.7_MA_0.05_Cs_0.25_Pb(I_0.8_Br_0.2_)_3_/**PDAI_2_**/C_60_/SnO_x_/Cu	1.243	20.9	82.7	21.48	*T*_96_ = 600 h@unencap., N_2_	[242]
1.68	ITP/PATT/FA_0.8_Cs_0.2_Pb(I_0.8_Br_0.2_)_3_:**DBF**/PCBM/BCP/Ag	1.2	20.51	82.04	20.18	*T*_90_ = 50 days@unencap., 30–40% RH, 25 °C, air	[160]
1.68	ITO/PTAA/2PACz/Cs_0.22_FA_0.78_Pb(I_0.8_Br_0.2_)_3_:**C‐βCD**/C_60_/BCP/Ag	1.15	22.53	80.4	20.83	n.a.	[280]
1.68	ITO/MeO‐2PACz/**CsPbCl_3_**/Cs_x_FA_1‐x_Pb(I_y_Br_1‐y_)_3_/LiF/C_60_/BCP/Ag	1.16	21.09	83.34	20.37	n.a.	[26]
1.68	ITO/NiO_x_/Me‐4PACz/FA_0.8_Cs_0.2_Pb(I_0.8_Br_0.2_)_3_/**BT2F‐2B**/C_60_/BCP/Ag	1.18	21.91	78.68	20.35	n.a.	[167]
1.68	ITO/SnO_2_/(FA, MA, Cs)Pb(I_0.7_Br_0.3_)_3_:**TOPO**/PTAA/Au	1.24	22.39	80.14	22.32	MPP, *T*_95_ = 1400 h@unencap., 100 mW cm^−2^ LED, 25 °C, N_2_	[159]
1.68	ITO/MeO‐2PACz/(FA, MA, Cs)Pb(I, Br)_3_:**TAACl**/CF_3_‐PEAI/C_60_/BCP/Ag	1.277	21.37	84.08	22.95	MPP, *T*_95_ = 1000 h@unencap., 100 mW cm^−2^ LED, 25 °C, N_2_	[165]
1.68	ITO/**β‐NapPAPT**/Cs_0.05_FA_0.8_MA_0.15_Pb(I_0.75_Br_0.25_)_3_/LiF/C_60_/BCP/Ag	1.21	20.91	85.50	21.63	MPP, *T*_98_ = 1000 h@unencap., 1‐sun LED, 25 °C, N_2_; *T*_91_ = 1000 h@encap., 50% RH, 65 °C, day/night cycle	[281]
1.68	ITO/Cs_0.05_FA_0.80_MA_0.15_PbI_2.25_Br_0.75_ (DMSO:ACN:EtOH green solvent)/C_60_/BCP/Cu	1.24	20.6	84	21.5	n.a.	[181]
1.68	ITO/2PACz/CS_0.22_FA_0.78_Pb(I_0.85_Br_0.15_)_3_:MAPbCl_3_/**PTFSI**/C_60_/SnO_x_/Ag	1.28	20.2	83.1	21.5	MPP, *T*_96_ = 1000 h@25 °C, N_2_, day/night cycle	[254]
1.685	ITO/SnO_2_/FA_0.8_Cs_0.2_Pb(I_0.75_Br_0.25_)_3_:**RbI**/FAI/**TOPO**/Spiro‐OMeTAD/Au	1.282	22.43	81.2	23.35	*T*_94_ = 3400 h@unencap., 10% RH, 25 °C, air; MPP, *T*_85_ = 3000 h@uncap., 100 mW cm^−2^ LED, 40 °C, N_2_; *T*_66.1_ = 280 h@unencap., 65 °C, N_2_; *T*_88_ = 350 h@encap., 30–40% RH, air	[125]
1.69	IZO/MeO‐4PACz/FA_0.8_MA_0.15_Cs_0.05_Pb(I_0.76_Br_0.24_)_3_/LiF/EDAI_2_/C_60_/IZO/MgF_2_	1.27	21.63	80.8	22.2	n.a.	[44]
1.7	FTO/TiO_2_/CsPbI_3_:**AFMS**/Spiro‐OMeTAD/Au	1.26	20.84	82.82	21.85	MPP, *T*_94_ = 550 h@unencap., 1‐sun, N_2_; *T*_92_ = 700 h@unencap., 30–40% RH, air	[153]
1.7	FTO/TiO_2_/CsPbI_3_/Spiro‐OMeTAD:**BPFPDS**/Au	1.29	20.80	81.92	21.95	MPP, *T*_98_ = 1200 h@encap., 100 mW cm^−2^, 30% RH, air; *T*_96_ = 3000 h@unencap., 15–20% RH, air; *T*_89_ = 300 h@unencap., 65 °C, N_2_	[198]
1.7	ITO/2PACz/(FA_0.7_MA_0.25_Cs_0.05_)Pb(I_0.75_Br_0.25_)_3_:**KSCN**/GuaI/EDA/C_60_/BCP/Cu	1.231	21	83.36	21.54	n.a.	[139]
1.7	ITO/SnO_2_/**Rb_0.04_Cs_0.2_FA_0.532_MA_0.228_Pb(I,Br,Cl)_3_**/Spiro‐OMeTAD/Au	1.24	20.6	80.5	20.6	MPP, *T*_92_ = 1000 h@unencap., 1‐sun, RT, N_2_	[282]
1.7	FTO/TiO_2_/CsPbI_3_:**PZD(BF_4_)_2_**/Carbon	1.13	20.47	79	18.27	*T*_65_ = 500 h@unencap., 85 °C, N_2_; MPP, *T*_80.6_ = 60 h@encap., 100 mW cm^−2^, air	[150]
1.7	ITO/NiO_x_/SAMs/**NCL**/CsPbI_3_/PCBM/BCP/Ag	1.25	20.5	83.1	21.24	*T*_97.61_ = 1000 h@unencap., 35% RH, air; MPP, *T*_92.27_ = 1000 h@encap., 100 mW cm^−2^	[283]
1.7	ITO/SnO_2_/Cs_0.03_(FA_0.9_MA_0.1_)_0.97_Pb(I_0.7_Br_0.3_)_3_ (by **Anti‐solvent IPA:PEACl**)/Spiro‐OMeTAD/Au	1.3	21.1	72.6	19.8	*T*_97_ = 1000 h@unencap., AM1.5G and (50 mW cm^−2^) 365 nm UV, 10–30% RH	[176]
1.7	ITO/PEDOT:PSS/Me‐4PACz/CsPbI_3_:**EAL**/DAB/PCBM/C_60_/BCP/Ag	1.2	21.53	81.6	21.08	MPP, *T*_98_ = 600 h@1‐sun LED, 45 °C, N_2;_ T_95_ = 1650 h@N_2_; *T*_90_ = 140 h@60 °C, N_2_	[71]
1.71	FTO/TiO_x_/CsPbI_3_:**CSE**/Spiro‐OMeTAD/Au	1.273	20.86	82.04	21.8	*T*_90_ = 1500 h@unencap., 25 °C, 25% RH, air; MPP, *T*_97_ = 440 h@encap., AM1.5G	[72]
1.71	ITO/NiO_x_/PTAA/AlO_x_/**3‐MeOPEACl**/Cs_0.05_FA_0.70_MA_0.25_PbI_2.25_Br_0.75_/PEAI/C_60_/SnO_x_/Ag	1.22	20.57	83	20.83	n.a.	[92]
1.71	FTO/TiO_2_/CsPbI_3_/**Tchl**/Carbon	1.146	19.08	81.64	19.08	MPP, *T*_93.2_ = 100 h@encap., 1‐sun LED, 65% RH, air; *T*_91.5_ = 300 h@unencap., 65 °C, N_2_	[284]
1.71	ITO/SnO_x_/CsPbI_2.85_Br_0.15_:**PC**/Spiro‐OMeTAD/Au	1.34	n.a.	n.a.	22.07	*T*_90.1_ = 1000 h@unencap., 65 °C, N_2_; MPP, *T*_90.89_ = 450 h@encap., air	[161]
1.71	ITO/NiO_x_/P3CT‐N/CsPbI_2.85_Br_0.15_/**ABF**/PCBM/BCP/Ag	1.247	19.62	84.97	20.8	*T*_94.2_ = 1200 h@25 °C, N_2_; *T*_89.83_ = 500 h@85 °C, N_2_; *T*_91.53_ = 500 h@20–25 °C, 15–25% RH, air; MPP, *T*_92.57_ = 200 h@unencap., 1‐sun LED, 25 °C, 20–30% RH, air	[258]
1.72	ITO/NiO_x_/MeO‐2PACz/FA_0.84_Cs_0.12_Rb_0.04_Pb(I_0.63_Br_0.37_)_3_/**PZDI**/C_60_/BCP/Ag	1.321	19.32	76.1	19.42	n.a.	[252]
1.73	ITO/SnO_x_/Cs_0.2_FA_0.8_Pb(I_0.7_Br_0.3_)_3_/**GuaCl**/Spiro‐OMeTAD/Ag	1.27	19.6	77.28	19.36	*T*_84_ = 1000 h@unencap., 40% RH, air; *T*_71_ = 250 h@unencap., 85 °C, N_2_	[240]
1.73	ITO/4PADCB/FA_0.8_Cs_0.2_Pb(I_0.7_Br_0.3_)_3_ (by **Close Space Annealing** with F‐PEACl:IPA)/F‐PEACl/C_60_/SnO_x_/ITO	1.299	18.80	82.9	20.24	n.a.	[185]
1.73	FTO/NiO_x_/MeO‐2PACz/CsPbI_3‐x_Br_x_/PCBM/BCP/Ag	1.256	20.38	83.6	21.4	MPP, *T*_90_ = 1260 h@encap., 1‐sun, RT, N_2_; T_93_ = 1000 h@unencap., 20–25% RH, RT, air; *T*_86_ = 350 h@unencap., 65 °C, N_2_	[285]
1.73	ITO/MeO‐2PACz/FA_0.8_Cs_0.2_Pb(I_0.7_Br_0.3_)_3_/**CCl**/C**_60_**/BCP/Cu	1.27	19.15	81.89	19.97	n.a.	[182]
1.74	ITO/PTAA:F4‐TCNQ/Cs_0.12_MA_0.05_FA_0.83_Pb(I_0.6_Br_0.4_)_3_ (by **DE:DMSO:MDF**)/PCBM/BCP/Ag	1.25	19.7	79.9	20.1	*T*_85_ = 16 days@unencap., 25 °C, 30% RH, air	[286]
1.75	FTO/NiO_x_/VNPB/Cs_0.42_DMA_0.28_MA_0.3_PbI_2.65_Br_0.35_ (by **anti‐solvent‐free**)/PCBM/BCP/Ag	1.184	20.39	81.78	19.76	n.a.	[41]
1.75	ITO/NiO_x_/**D‐2P**/FA_0.8_Cs_0.15_MA_0.05_Pb(I_0.7_Br_0.3_)_3_/PEAI/C_60_/BCP/Ag	1.32	18.81	83.59	20.8	*T*_90+_ = 2500 h@unencap., 1‐sun, N_2_	[287]
1.77	ITO/FA_0.8_Cs_0.2_Pb(I_0.6_Br_0.4_)_3_:(**MeO‐2PACz:Me‐4PACz**)/p‐F‐PEAI/C_60_/BCP/Ag	1.313	18.19	80.84	19.31	MPP, *T*_85_ = 260 h@unencap., AM1.5G, N_2_	[154]
1.77	ITO/Me‐4PACz/ FA_0.8_Cs_0.2_Pb(I_0.6_Br_0.4_)_3_/**SEBr**/LiF/C_60_/BCP/Ag	1.33	18.27	82.06	19.9	n.a.	[257]
1.77	ITO/**DCB‐BPA**/FA_0.8_Cs_0.2_Pb(I_0.6_Br_0.4_)_3_:Pb(SCN)_2_/TEACl/C_60_/BCP/Cu	1.33	17.75	82.7	19.53	*T*_80_ = 417 h@encap., 100 mW cm^−2^ LED, ≈50% RH	[206]
1.77	ITO/NiO_x_/Me‐4PACz/Cs_0.2_FA_0.8_Pb(I_0.6_Br_0.4_)_3_:MACl (by **Blade coating**)/TEACl/C_60_/BCP/Ag)	1.3	18.07	83.21	19.55	MPP, *T*_90_ = 610 h@AM1.5G, N_2_	[218]
1.77	ITO/Me‐4PACz/FA_0.85_Cs_0.15_Pb(I_0.6_Br_0.4_)_3_/**AEAPTMS**/PCBM/BCP/	1.35	17.2	80.9	18.7	n.a.	[43]
1.77	ITO/2FMPA‐BT‐BA/FA_0.8_Cs_0.2_Pb(I_0.6_Br_0.4_)_3_:Pb(SCN)_2_/**2‐MePBr**/C_60_/SnO_x_/Cu	1.29	18.12	83.08	19.42	MPP, *T*_80_ = 337 h@encap., 1‐sun, air; *T*_83_ = 262 h@encap., 55 °C, air; *T*_75_ = 25 days@unencap., 30 °C, 45–48% RH, air	[255]
1.77	ITO/NiO_x_/SAM/DMA_0.1_Cs_0.3_FA_0.6_Pb(I_0.7_Br_0.3_)_3_ (by **Gas quenching**)/TEACl/PCBM/AZO NPs/SnO_x_/Ag	1.244	17.9	82.1	18.28	MPP, *T*_95_ = 900 h@unencap., 1‐sun WLED, 55 °C, N_2_; *T*_90_ = 800 h@85 °C	[183]
1.77	ITO/NiO_x_/2PACz/FA_0.8_Cs_0.2_Pb(I_0.6_Br_0.4_)_3_/**FEDA**/C_60_/BCP/Ag	1.34	18.16	83.62	20.37	MPP, *T*_97_ = 800 h@1‐sun LED, 25 °C, air	[250]
1.77	MgF_2_/ITO/NiO_x_/2PACz:MeO‐2PACz/FA_0.83_Cs_0.17_Pb(I_0.6_Br_0.4_)_3_/PEAI:MAI/C_60_/**YbO_x_**/Cu	1.3	18.6	93	20.1	*T*_80_ = 500 h@AM1.5 G, 85 °C, N_2_	[49]
1.77	ITO/4PADCB/FA_0.8_Cs_0.2_Pb(I_0.6_Br_0.4_)_3_/**4‐ATpHCl**/C_60_/BCP/Cu	1.314	17.65	83.32	19.32	MPP, *T*_80_ = 519 h@encap., 1‐sun, air; *T*_90_ = 220 h@unencap., 55 °C, N_2_	[256]
1.77	ITO/NiO_x_/Me‐4PACz/ FA_0.8_Cs_0.2_Pb(I_0.6_Br_0.4_)_3_:**DDPA**/PDAI_2_/C_60_/BCP/SnO_x_/Cu	1.32	18.09	84.4	20.2	*T*_85_ = 16 days@unencap., 25 °C, 30–80% RH, air; MPP, *T*_80_ = 500 h@unencap., 1‐sun, 55 °C, N_2_	[158]
1.77	ITO/MeO‐2PACz/FA_0.8_Cs_0.2_Pb(I_0.6_Br_0.4_)_3_/**PDADI:FAI**/C_60_/BCP/Cu	1.296	18.04	81.01	19.52	MPP, *T*_109_ = 400 h @unencap., 100 mW cm^−2^ LED, 50 °C, N_2_	[241]
1.77	ITO/NiO_x_/MeO‐2PACz/FA_0.8_Cs_0.2_Pb(I_0.6_Br_0.4_)_3_/**EDA:SnO_2_ NPs**/PEIE/Ag	1.25	18.82	80.3	18.9	*T*_97_ = 1340 h@encap., 20% RH, 20–25 °C, air	[288]
1.77	ITO/NiO_x_/2PACz:MeO‐2PACz/FA_0.8_Cs_0.2_Pb(I_0.6_Br_0.4_)_3_/**F‐PEACl:CF3‐PACl**/C_60_/SnO_x_/Cu	1.36	18.3	82.3	20.5	MPP, *T*_100_ = 200 h@encap., AM1.5G, 65 °C, 20–30% RH, air	[45]
1.77	ITO/MeO‐2PACz/FA_0.8_Cs_0.2_Pb(I_0.6_Br_0.4_)_3_:**GlyACl**/C_60_/BCP/Cu	1.31	18.23	83.59	19.97	MPP, *T*_92_ = 500 h@unencap., AM1.5G, 55 °C, N_2_	[164]
1.77	ITO/4PADCB/FA_0.8_Cs_0.2_Pb(I_0.6_Br_0.4_)_3_:I**A**/EDAI_2_/C_60_/BCP/Ag	1.342	17.39	82.9	19.34	MPP, *T*_90_ = 700 h@encap., 100 mW cm^−2^ LED; *T*_88_ = 300 h@100 mW cm^−2^ LED, 65 °C	[166]
1.78	ITO/NiO_x_/Me‐4PACz/FA_0.65_Cs_0.35_Pb(I_0.6_Br_0.4_)_3_ (**DMSO:ACN:EtOH** green solvent)/C_60_/BCP/Cu	1.31	18.3	82.3	19.6	n.a.	[181]
1.78	ITO/NiO_x_/2PACz/FA_0.8_Cs_0.2_Pb(I_0.6_Br_0.4_)_3_/**F‐PEAI**/C_60_/BCP/Ag	1.35	17.52	82	19.4	*T*_92_ = 500 h@AM1.5G	[50]
1.78	ITO/NiO_x_/Me‐4PACz/FA_0.8_Cs_0.2_PbI_1.8_Br_1.2_:**BT2F‐2B**/C_60_/SnO_x_/Ag	1.31	17.99	84.18	19.78	n.a.	[167]
1.78	ITO/2PACz/(FA_0.6_MA_0.4_)Pb(I_0.6_Br_0.4_)_3_:**KSCN**/GuaI/EDA/C_60_/BCP/Cu	1.284	18.96	84.03	20.45	n.a.	[139]
1.78	ITO/2PACz:MeO‐2PACz/FA_0.8_Cs_0.2_Pb(I_0.6_Br_0.4_)_3_ (**MAI:PEA:IPA:Anisole antisolvent)**/EDAI_2_/C_60_/SnO_x_/Ag	1.373	18.1	84.7	21.1	MPP, *T*_90_ = 500 h@100 mW cm^−2^ AM1.5G; *T*_85_ = 500 h@85 °C	[289]
1.78	ITO/NiO_x_/Me‐4PACz/Cs_0.2_FA_0.8_Pb(I_0.63_Br_0.37_)_3_/**5‐AVAI/AlO_x_**/C_60_/SnO_x_/Ag	1.311	18.3	83.1	19.9	MPP, *T*_90_ = 1615 h@encap., 1‐sun, 25–55 °C, air	[272]
1.79	ITO/NiO_x_/MeO‐2PACz/ FA_0.8_Cs_0.2_Pb(I_0.6_Br_0.4_)_3_:**PI**/C_60_/BCP/Ag	1.332	17.37	82.9	19.59	n.a.	[38]
1.79	ITO/PTAA/2PACz/Cs_0.4_FA_0.6_Pb(I_0.65_Br_0.35_)_3_:**C‐βCD**/C_60_/BCP/Ag	1.21	17.27	76.8	16.05	MPP, *T*_93.7_ = 1200 h@encap., 100 mW cm^−2^ AM1.5G; *T*_95_ = 2800 h@encap., 30% RH, air; *T*_82_ = 520 h@unencap., 65 °C, N_2_	[280]
1.79	ITO/**DCB‐Br‐2**/FA_0.8_Cs_0.2_Pb(I_0.6_Br_0.4_)_3_/EDAI_2_/C_60_/BCP/Ag	1.37	18.12	83.53	20.76	n.a.	[208]
1.79	ITO/NiO_x_:**SHMP/**Me‐4PACz/FA_0.8_Cs_0.2_Pb(I_0.6_Br_0.4_)_3_/PDADI/C_60_/BCP/Ag	1.348	17.87	87.27	21.02	MPP, *T*_80_ = 620 h@encap., 1‐sun, 34 °C, N_2_; *T*_86_ = 980 h@encap., 70–80% RH, air; *T*_92_ = 800 h@encap., 60 °C, N_2_	[290]
1.79	ITO/CbzNaph/Cs_0.2_FA_0.8_Pb(I_0.6_Br_0.4_)_3_:/C_60_/BCP/Ag	1.35	17.29	83.07	19.5	MPP, *T*_93_ = 500 h@encap., AM1.5G, 45 °C, N_2_	[121]
1.8	ITO/NiO_x_/N719/Cs_0.35_FA_0.65_Pb(I_0.6_Br_0.4_)_3_:**MDI‐PU**/C_60_/BCP/Ag	1.274	17.4	79.6	17.6	*T*_85.5_ = 120 days@encap., N_2_	[238]
1.81	ITO/DC‐PA FA_0.8_Cs_0.2_Pb(I_0.6_Br_0.4_)_3_:GBAC:**AQSP**/PI/C_60_/BCP/Ag	1.351	17.52	82.74	19.58	MPP, *T*_95_ = 500 h@encap., AM1.5G, N_2_	[39]
1.82	ITO/2PACz/Cs_0.05_(FA_0.55_MA_0.45_)_0.95_Pb(I_0.55_Br_0.45_)_3_/**CCI** or **LiF**/C_60_/BCP/Ag	1.21	15.9	77	14.6	n.a.	[33]
1.82	ITO/2PACz/(FA_0.5_MA_0.45_Cs_0.05_)Pb(I_0.55_Br_0.45_)_3_:**KSCN**/GuaI/EDA/C_60_/BCP/Cu	1.305	17.36	84.82	19.22	MPP, *T*_85_ = 250 h@unencap., 1‐sun, 30 – 50% RH, air; *T*_92_ = 5000 h@unencap., N_2_	[139]
1.83	ITO/NiO_x_/2PACz/ FA_0.8_Cs_0.2_PbI_1.6_Br_1.4_:**Pb(SCN)_2_**/PEAI/C_60_/BCP/Ag	1.32	17.06	84.21	18.96	MPP, *T*_91_ = 1000 h@100 mW cm^−2^ LED, 45 – 55 °C, N_2_	[36]
1.85	FTO/SnO_2_/Cs_0.17_FA_0.83_PbI_1.5_Br_1.5_:**GABr**/Spiro‐OMeTAD/Au	1.258	14.3	71.8	12.9	n.a.	[67]
1.86	ITO/NiO_x_/Me‐4PACz/Cs_0.25_FA_0.75_Pb(Br_0.5_I_0.5_)_3_:**AIDCN**/PCBM/BCP/Ag	1.366	16.10	84.2	18.52	MPP, *T*_80_ = 271 h@encap., 1‐sun, 25 °C, 85% RH, air	[162]
1.88	FTO/Me‐4PACz/FA_0.7_MA_0.2_Rb_0.1_Pb(I_0.5_Br_0.5_)_3_/**cis‐CyDAI2**/C60/BCP/Ag	1.36	16.1	83.8	18.1	MPP, *T*_86_ = 450 h@encap., 1‐sun, air	[291]
1.91	ITO/ZnO/CsPbI_2_Br:**ZrCl_4_**/Spiro‐OMeTAD/Ag	1.29	15.84	81.14	16.6	*T*_91_ = 1000 h@unencap., AM1.5G, RT, 20% ± 5% RH, air; *T*_92_ = 500 h@85 °C, N_2_	[292]
1.91	FTO/c‐TiO_2_/**AIV NSs**/CsPbI_2_Br/Carbon	1.31	14.78	77.9	15.25	MPP, *T*_80_ = 240 h@25 °C, 50% RH, air; *T*_80_ = 1440 h@unencap., 25 °C, 10% RH, air; *T*_80_ = 740 h@unencap., 85 °C, 20%RH	[293]
1.91	ITO/**Cl@MZO**/CsPbI_2_Br/PM6/MoO_3_/Ag	1.305	21.9	81	18.6	*T*_94.1_ = 500 h@unencap., 25 °C, 10–15% RH, air	[199]
1.93	ITO/NiO_x_/Me‐4PACz/FA_0.6_MA_0.15_Cs_0.25_Pb(I_0.45_Br_0.5_ **OCN**_0.05_)_3_/PCBM/BCP/Ag	1.422	14.18	93.79	16.9	MPP, *T*_96_ = 300 h@encap., 1‐sun, 25 °C, >60% RH, air	[35]
1.96	ITO/NiO_x_/2PACz/**Rb_0.05_Cs_0.12_FA_0.83_PbI_1−x_Br_2_Cl_x_**/LiF/C_60_/BCP/Ag	1.33	13.05	76.7	13.4	n.a.	[137]
1.97	ITO/NiO_x_/Me‐4PACz/Cs_0.15_MA_0.85_Pb(I_0.1_Br_0.9_)_3_:**PDAI_2_**/**PDAI_2_**/PCBM/BCP/Ag	1.44	12.8	0.83	15.3	MPP, *T*_90_ = 20 h@encap., AM1.5G, RT	[293]
1.99	ITO/PTAA/2PACz/Cs_0.2_FA_0.8_Pb(I_0.3_Br_0.7_)_3_:**C‐βCD**/C_60_/BCP/Ag	1.17	11.13	66.3	8.63	n.a.	[280]
2.1	FTO/c‐TiO_2_/**AIV NSs**/CsPbIBr_2_/Carbon	1.316	11.79	73.6	11.42	n.a.	[293]

The strategies adopted in each corresponding study are marked in bold; *T*_number_ means the number% efficiency retention under different stability testing conditions; encap. is the abbreviation of encapsulation; unencap. is the abbreviation of un‐encapsulation; MPP represents efficiency tracking at maximum power point; RH stands for relative humidity; NPs denote nano‐particles; NSs signify nano‐sheets.

### 3.1 Bulk Passivation

#### 3.1.1 Composition Regulation

Given that photo‐induced I‐Br phase segregation tends to occur at high Br content ratios (>20%), a straightforward solution is to reduce or eliminate the incorporation of bromine. Previous successful practical implementations have involved the introduction of large‐sized organic cations, such as Dimethylammonium (DMA^+^, 272 pm),^[^ ^41^, ^82^, ^114^, ^115^ ^]^ Guanidinium (GA^+^, 278 pm),^[^ ^116^ ^]^ Methylenediammonium (MDA^2+^, 230 pm),^[^ ^117^, ^118^ ^]^ Ethylammonium (EA^+^, 274 pm),^[^ ^119^ ^]^ and Imidazolium (IA^+^, 258 pm),^[^ ^120^, ^121^ ^]^ etc. Their relatively appropriate molecule sizes enable them to successfully entry into the cavities composed of perovskite octahedra, while expanding lattice and tuning bandgaps. For example, in terms of obtaining perovskites with an equivalent wide bandgap, adding 10 mol% DMA^+^ into DMA_x_Cs_0.4_FA_0.6‐x_Pb(I_1‐y_Br_y_)_3_ could dramatically decrease the bromine proportion from 0.4 to 0.25, leading to over a two‐fold increase in photostability for 90% efficiency retention under continuous illumination.^[^ ^122^ ^]^ These organic cations not only positively relax lattice strains and suppress defect generation, but also compensate for tolerance factors to enhance perovskite phase stability. Furthermore, they are benign for the crystallization growth of perovskites. Tang's team disclosed the orientational induction effect of 1D‐DMAPbI_3_ nuclei on thermally evaporated 1.73 eV‐CsPbI_3_, beneficial for increasing grain size, enhancing crystallinity, and a nearly fivefold extension of carrier lifetime. The resultant devices set a record efficiency of 16.1% and a high *V*_oc_ of ≈1 V, accompanied by a small non‐radiative recombination voltage loss below 0.3 V.^[^ ^123^ ^]^ Divalent MDA^2+^ with its stronger charge centers has been reported to intensify the solvation effect of aprotic polar solvents, facilitating the homogeneity of perovskite precursor by inhibiting the formation of intermediates that would lead to the aggregation or segregation of A‐site cations in FA‐Cs compounds during growth.^[^ ^117^, ^124^ ^]^ Additionally, due to the abundance of hydrogen atoms, organic cations can strongly combine with halogen ions through hydrogen bonding and consequently reduce the density of interstitial defects. By using photoemission electron spectroscopy to investigate sub‐gap states, Zhou et al. identified that GA^+^ was capable of contrapuntally passivate negative iodide interstitials (I_i_^−^) that are commonly regarded as hole traps responsible for the generation of neutral iodine radicals and I_2_. Eventually, *V*_oc_ escalated from 1.165 to 1.258 V as a result of the optimized carrier dynamics.^[^ ^67^ ^]^

**Figure 3:**
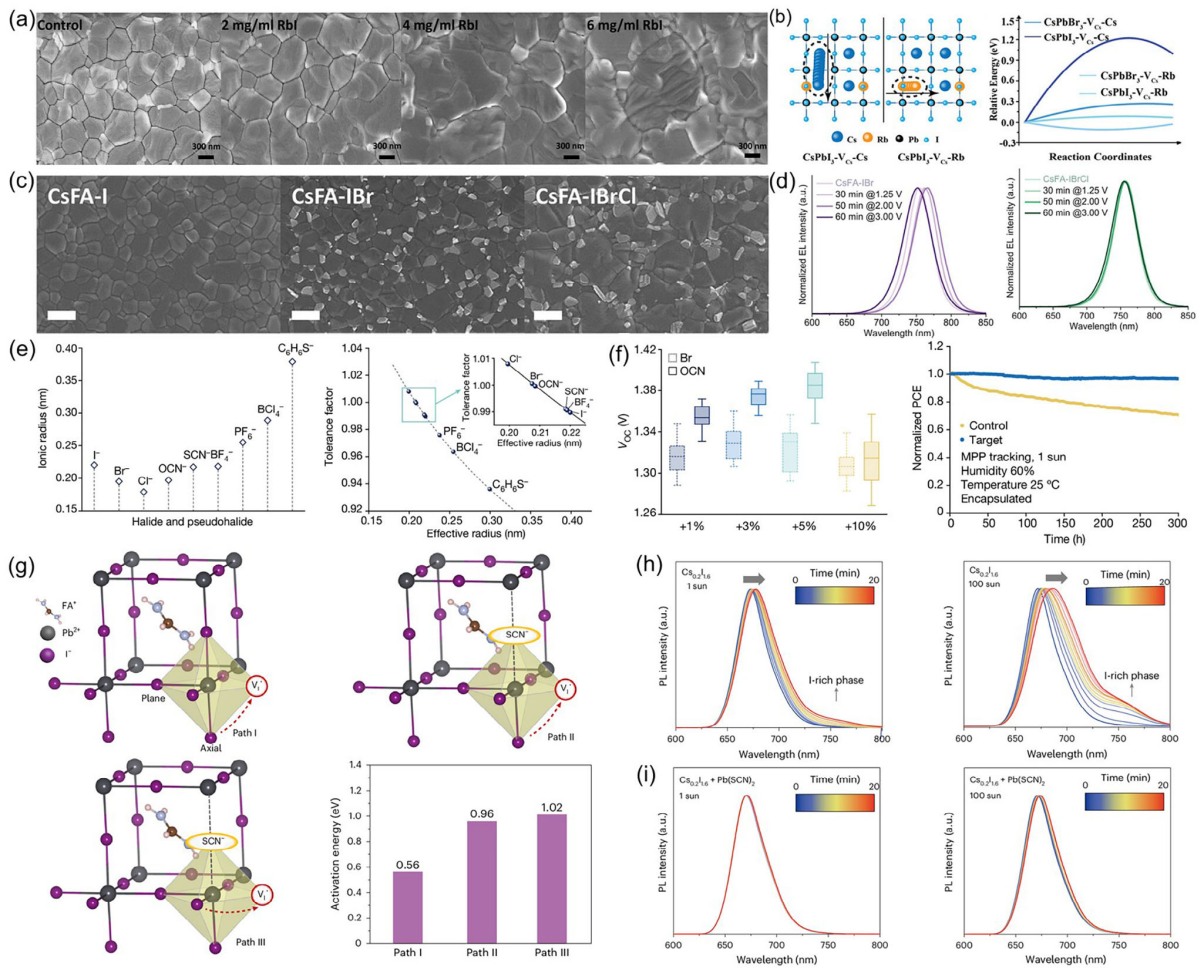
a) Top‐view SEM images of WBG perovskite films, revealing the effect of different concentrations of RbI doping on grain sizes. Reproduced with permission.^[^ ^125^ ^]^ Copyright 2024, The Royal Society of Chemistry. b) Computational model (left) and results (right) for evaluating the formation energy of interstitial Cs^+^ and Rb^+^ migration in perovskite lattices. Reproduced with permission.^[^ ^129^ ^]^ Copyright 2024, Wiley‐VCH GmbH. c) Top‐view SEM images of the morphologies of mono‐, binary‐, and ternary‐halide WBG perovskite films, as well as (d) the normalized electroluminescence spectral stabilities of the binary‐ and ternary‐halide devices at different voltage bias over long periods of time. c,d) Reproduced under the terms of the Creative Commons CC‐BY license (https://creativecommons.org/licenses/by/4.0/).^[^ ^75^ ^]^ Copyright 2024, The Authors, published by Wiley‐VCH GmbH. e) Effective ionic radius (left) of halides and pseudohalides and the tolerance factor (right) of 5% pseudohalide‐substituted perovskite FA_0.75_Cs_0.25_Pb(I_0.5_Br_0.5_)_3_. Reproduced with permission.^[^ ^35^ ^]^ Copyright 2024, Springer Nature. f) Variation of *V*_oc_ with different doping concentrations of Br and OCN (left) and the long‐term operation stability of the pseudohalide OCN‐doped WBG PSCs under 1‐sun illumination (right). Reproduced with permission.^[^ ^35^ ^]^ Copyright 2024, Springer Nature. g) I^−^ migration pathways and the corresponding formation energies without/with SCN^−^ doped in FAPbI_3_ perovskites. Reproduced with permission^[^ ^36^ ^]^ Copyright 2024, Springer Nature. PL evolution of WBG perovskites without (h) and with (i) SCN^−^ alloyed under 1‐sun (left) and 100‐sun (right) illumination for 20 min. h,i) Reproduced with permission.^[^ ^36^ ^]^ Copyright 2024, Springer Nature.

Another effective approach to strengthen the operational stability and *V*_oc_ of WBG perovskite devices is to practice multi‐mixed A‐site cations or X‐site anions, as well as their shared utilization. Ye et al. observed the more accrescent grains with the higher concentration of rubidium iodide (RbI) doping in FA_0.8_Cs_0.2_Pb(I_0.75_Br_0.25_)_3_ (**Figure** **3**a), which significantly reduced grain boundaries and polished surfaces.^[^ ^125^ ^]^ Champion photovoltaic performances at an optimal amount of 4 mol% RbI were obtained for 20.11% PCE with 1.233 V *V*_oc_ in the regular devices and 21.31% PCE with 1.198 V *V*_oc_ in the inverted ones. In fact, a smaller ionic radius of Rb^+^ (152 pm), compared to Cs^+^ (167 pm) and FA^+^ (253 pm), guarantees its timely filling for A‐site cation vacancies so as to diminish defect states, mitigate lattice strain, and raise ion migration barriers.^[^ ^126^, ^127^, ^128^ ^]^ DFT calculations demonstrated that the much lower migration energies could drive interstitial Rb^+^ ions to occupy A‐site vacancies in an almost spontaneous manner (Figure 3b).^[^ ^129^ ^]^ Favorable improvement in microscopic morphologies can also be realized by introducing chloride ions. Saliba's team compared tuple halide CsFA‐I, binary halides CsFA‐IBr, and triplet halides CsFA‐IBrCl systems, finding a spatial increase in grain size (Figure 3c) and an improvement in photocarrier lifetimes with a mere 3% PbCl_2_ doping. Triple halide devices not only presented extraordinary endurance to bias voltages over a long period of time (Figure 3d), but also harvested cutting‐edge photovoltaic performance (22.6% PCE and 1.23 V *V*_oc_) in 1.64 eV WBG solar cells. After 1000 h tracking under ISOS protocols, targeted devices performed 95% efficiency retention and 74% thermal retention. Volatile MACl is also popularly regarded as the supply source of chlorine, which can produce salutary Cl‐rich intermediate phases to orient crystal growth, inhibit Br‐ and I‐rich transitional species to eliminate surface “wrinkles”, retard perovskite crystallization rates to promote compositional homogeneity and passivate halide defects to enhance phase stability.^[^ ^27^, ^41^, ^130^, ^131^, ^132^ ^]^ Studies have indicated that the residual chlorine content in perovskite films is closely related to the proportion of Br within systems.^[^ ^27^, ^133^, ^134^ ^]^ However, unlike Pb‐Cl blended compounds, the inclusion of MACl would have a negligible effect on bandgaps even in large quantities.^[^ ^129^, ^132^ ^]^ More crucially, an appropriate amount of MACl helps to clear up the residence of PbI_2_, thereby preventing potential defects and device degradation caused by its photolysis byproducts, such as Pb^0^ and I_2_.^[^ ^65^, ^132^, ^135^, ^136^ ^]^ Combined with Rb^+^ and Cl^−^, Li et al. boosted the *V*_oc_ of 1.96 eV devices to 1.33 V from 1.26 V with a significantly reduced hysteresis index of 0.06, attributed to well‐alloyed phases and suppressed non‐radiative decay.^[^ ^137^ ^]^ Special attention should be paid to other monovalent cations (e.g., Li^+^, Na^+^, K^+^, etc.) and halide anions (e.g. F^−^, etc.) that are too small to be integrated into perovskite lattice. Instead, they are energy‐preferentially distributed at grain boundaries and surfaces where they can either sequester halides or passivate uncoordinated Pb^2+^ to immobilize ions and defects, as well as reduce electron–phonon coupling through homogenized halides. Conclusively, they play a vital role in the modulation of crystallinity, optimization of charge carrier properties, diminishment of trap states, and photostability improvement.

Pseudohalogens, also known as halogen analogs, refer to the chemical species with comparable effective ionic radii, similar properties, and reactivity to halogens, which enable the formation of stable perovskite phases (Figure 3e). These anions are typically characterized by a high electronegativity, suggesting stronger interaction with [PbX_6_]^4−^ so as to increase the formation energy of perovskite intermediates and slow down crystallization rate. As a consequence, perovskite films with incorporated pseudohalides often exhibit larger grain sizes and superior crystal quality, effectively controlling the generation of defect sites. Very recently, linear cyanate (OCN^−^) has been confirmed to partially substitute bromine, as evidenced by the notable lattice distortion.^[^ ^35^ ^]^ OCN made halides distributed more uniformly and micro‐strain more consistent, resulting in a record *V*_oc_ of 1.422 V and a long‐term maximum power point (MPP) operation (*T*_96_ > 300 h) for 1.9‐eV ultra‐WBG solar cells (Figure 3f). Thiocyanate (SCN^−^), which is equivalent in size to I^−^ ions, is a linear Lewis alkaline substance with stable resonance and is one of the most commonly used additives in wide bandgap perovskites.^[^ ^138^, ^139^, ^140^ ^]^ In the past, SCN^−^ was highly believed to volatilize from perovskite films as gaseous HSCN products during annealing, possibly leaving residues far below the detectable resolution.^[^ ^141^, ^142^, ^143^ ^]^ However, Zhang et al. recently provided indirect evidence for the presence of trace amounts of SCN^−^ within the grown thin film via a chromogenic reaction involving FeCl_3_, which was further supported by Fourier transform infrared and X‐ray photoelectron spectroscopy characteristics.^[^ ^36^ ^]^ Also, through DFT calculations, it was proposed that SCN^−^ could readily be incorporated into the iodoplumbate complex by coordinating with cations, forming ternary I/Br/SCN alloys. The occupation of SCN^−^ in I‐site increased the I^−^ migration energy along paths between axes and planes (Figure 3g), thus notably suppressing the photo‐induced phase segregation under 1‐sun to 100‐sun illumination (Figure 3h,i). Apart from the above candidates, BF_4_^−^ and slightly larger PF_6_^−^ have also been demonstrated to not only counteract phase separation through lattice stress release and halogen vacancies suppression, but also finely tune the optical bandgap and Fermi level to strengthen the built‐in electric field and promote carrier transport and collection.^[^ ^144^, ^145^, ^146^ ^]^

In summary, compositional engineering presents a fundamental avenue for optimization of performance and enhancement of stability in WBG PSCs. Through strategic manipulation of composition, substantial advancements can be attained in tolerance factor matching, crystal growth modes, microstructural morphology, and charge carrier properties. These enhancements collectively contribute to a reduction of non‐radiative recombination losses in *V*_oc_. Moreover, compositional engineering augments ion migration activation energy and alleviates lattice strain, thereby fortifying the stability of perovskite phases under high‐voltage and high‐illumination conditions.

#### 3.1.2 Additive Engineering

The main purpose of additive engineering is to modulate the crystallization growth kinetics among mixed perovskite components, expected to fabricate the high‐quality films with large grains, free‐of‐pinholes, uniform, smoothness, phase homogeneity, and low defect state density. Several classes of additives that have been demonstrated to dramatically upgrade the performance and stability of WBG PSCs are going to be highlighted as follows.

**Figure 4:**
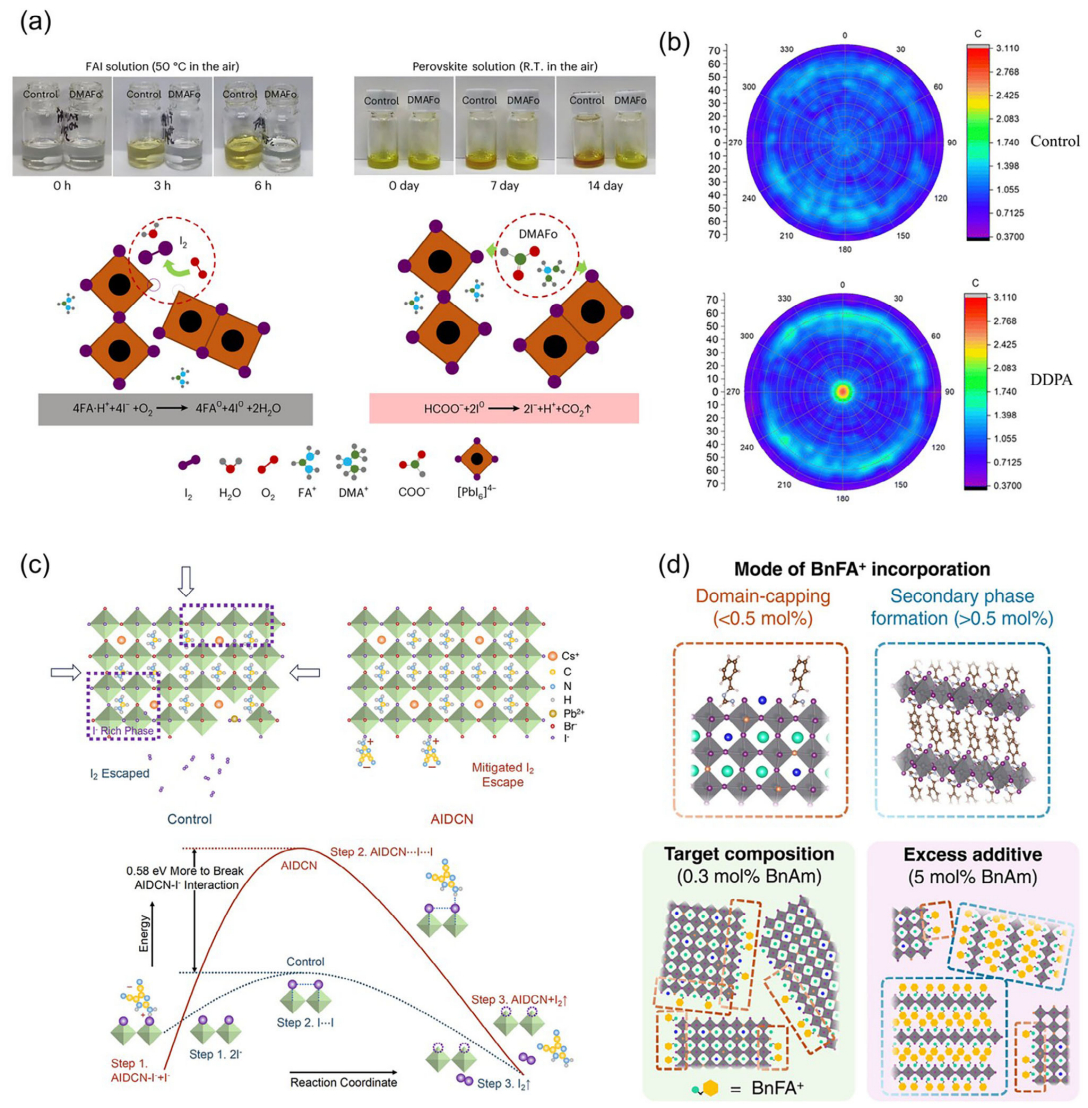
a) The color‐changing photographs of samples without/with DMAFo stabilizer, aged in ambient air at 50 °C, accompanied by the underlying oxidation and reduction reactions. Reproduced with permission.^[^ ^37^ ^]^ Copyright 2024, Springer Nature. b) Pole figures of the (011) reflections at 2*θ* = 20.3° in control and DDPA‐treated perovskite films. Reproduced with permission.^[^ ^158^ ^]^ Copyright 2024, The Royal Society of Chemistry. c) Sketch illustrating halide phase segregation and iodine escape in control and AIDCN‐doped perovskite films (Top) and their calculated energy diagrams of the iodine escape process (Bottom). Reproduced with permission.^[^ ^162^ ^]^ Copyright 2024, Elsevier. d) Schematics of two possible additive concentration‐dependent modes of BnFA^+^ incorporation into perovskite materials. Reproduced under the terms of the CC‐BY Creative Commons Attribution 4.0 International License (https://creativecommons.org/licenses/by/4.0/).^[^ ^168^ ^]^ Copyright 2024, The Authors, published by American Chemical Society.

Ionic liquids, attractive for their low volatility, thermal stability, hydrophobicity, strong chemical tunability, and eco‐friendliness, have been extensively applied to perovskites.^[^ ^147^, ^148^ ^]^ As a class of molten salts, ionic liquids composed of organic cations (e.g., imidazole, pyridine, pyrrolidine) and inorganic or organic anions (e.g., halides, formate, acetate, tetrafluoroborate, hexafluorophosphate).^[^ ^37^, ^145^, ^149^, ^150^, ^151^, ^152^ ^]^ They impede nucleation and retard crystallization via interacting with perovskites by hydrogen bonds, chelation bonds, or forming intermediates. Xu's team proposed a counter‐ion designed DMAFo and evaluated its ability as an additive to prepare PSCs in ambient.^[^ ^37^ ^]^ It was found that DMA^+^ promoted the multistage intermediate phases, while the reducible ─COOH bonded iodide ions via H⋯I and prevented organic cations from deprotonation (**Figure** **4**a). Devices optimized at 1.65 eV perovskites maintained its initial MPP efficiency without loss following accelerated aging at 85 °C for 1000 h. This could be attributed to the reduced intra‐bandgap defect states and elevated ion mobility activation energy. Pei et al. modified the alkyl chain of 1‐butyl‐1‐methylpiperidinium (BMP) cations with an extra methoxyethyl group to synthesize a new 1‐(2‐methoxyethyl)‐1‐methylpiperidinium (MMP)‐based compound.^[^ ^149^ ^]^ MMP coordinated with Pb^2+^ more strongly so that deep‐level defects were effectively suppressed in bulks. Strikingly, Yang et al. proposed a flowing liquid‐phase methodology that capitalizes on the low decomposition temperature of NH_4_COOH. This approach induced a solid‐to‐liquid transition during the annealing process of perovskite thin films, facilitating crystallization while liberating functional ions to prevent the formation of non‐perovskite phases by I^−^ and Pb^2+^ in CsPbI_3_ system.^[^ ^153^ ^]^

The phosphate functional group provides strong binding sites that effectively interact with lead cations, offering a promising avenue for passivating deep‐level defects in perovskites. Recent studies have underscored the remarkable performance enhancements by employing carbazole‐ and pentafluorobenzyl‐based phosphonic acid compounds in WBG PSCs.^[^ ^48^, ^154^, ^155^, ^156^, ^157^ ^]^ In a typical bottom‐up crystallization mode, these oversized and negatively polarized molecules are extruded onto the upper and buried surfaces, as well as grain boundaries, where they act on the uncoordinated Pb^2+^ and Pb^0^ defects through Pb─O bonds.^[^ ^155^, ^156^, ^157^ ^]^ On top of that, the carbazole groups containing N atoms with lone electron pairs and adjunct functional groups (e.g. methyl, methoxy, bromine atoms) can further engage with other defective species, so do the fluorobenzyl ones. Noteworthy for their large dipole moments, these molecules wield great influence over the energy level modulation of the perovskites and even the substrates according to the sediment‐dispersed modifiers. Very recently, Guan et al. reported a dodecylphosphonic acid (DDPA) material, in which the elongated alkyl chain promoted the vertical growth of grains, favoring (011) crystallographic orientation (Figure 4b).^[^ ^158^ ^]^ By inhibiting the formation of vacancies, the implementation of DDPA yielded exceptional 20.2% efficiency for perovskites characterized by a bandgap of 1.77 eV. Yao et al. suggested that all‐phosphate‐containing materials possess the capability to control crystal growth through coordination with Pb^2+^ cations, where the growth rate is determined by the branched chain. By introducing trioctylphosphine oxide (TOPO), they successfully synthesized (111)‐plane‐oriented growth of 1.68 eV perovskites, leading to improved charge‐carrier tolerance and attaining a T95 stability of over 1000 h for MPP tracking.^[^ ^159^ ^]^

Lewis acid/base additives are beneficial to delay crystallization, release film strain, coordinate Pb^2+^ or I^−^, and inhibit ion migration.^[^ ^74^, ^160^, ^161^, ^162^, ^163^ ^]^ Organic molecules assembled with ligands or groups having Lewis acid/base functions, such as pyridine,^[^ ^161^ ^]^ acridine,^[^ ^71^ ^]^ thiophene,^[^ ^163^ ^]^ furan,^[^ ^160^ ^]^ amide,^[^ ^161^, ^164^, ^165^, ^166^ ^]^ and lactic acid,^[^ ^71^ ^]^ form adducts with perovskites at grain boundaries and surfaces. These tailored chelates bolster the crystalline uniformity and intensity while establishing hydrophobic wrapping shells to prevent moisture intrusion. The spatial arrangement of conjugated backbones and π–π stacking in some organic molecules play a critical role in facilitating charge transport, thus enhancing the dynamic behavior of charge carriers.^[^ ^71^, ^160^ ^]^ To address the challenge of phase segregation caused by photo‐induced iodine diffusion, Hou's research group introduced 2‐amino‐4,5‐imidazoledicarbonitrile (AIDCN) with high dipole moments into the 1.86 eV perovskite materials, which strengthened the chemical anchoring between amino groups and iodides. AIDCN also raised the activation energy required for iodine escape, and mitigated lattice shrinkage (Figure 4c).^[^ ^162^ ^]^ A record voltage of 1.366 V was finally achieved, and a photostability duration was prolonged, nearly doubling that of the control. Very recently, Song et al. designed an electrically neutral Lewis acid, 2,1,3‐benzothiadiazole,5,6‐difluoro‐4,7‐bis(4,4,5,5‐tetramethyl‐1,3,2‐dioxaborolan‐2‐yl) (BT2F‐2B), to strongly immobilize I^−^ through its unhybridized p‐orbitals and electronegative fluorine ions, thereby impeding a transition to I_2_. This additive is universally applicable, realizing high efficiencies of 19.78% and 20.35% in 1.77 eV and 1.67 eV PSCs, respectively.^[^ ^167^ ^]^

Concerning other types of additives, Yang et al. harnessed the dual functionality in chlorinated silane molecules, where chloride ions assisted in crystallization and passivated Pb‐related defects, while the silane moiety with hydrolytic polymerization ability spontaneously constructed a moisture barrier.^[^ ^72^ ^]^ The photo‐voltage of CsPbI_3_ devices was notably boosted to 1.27 V, alongside an exceptional 90% efficiency retention in unencapsulated samples subjected to a 1500‐h air storage. Zhou et al. proposed a novel in‐situ reaction mechanism between amine‐based additives and the organic cations present in perovskites.^[^ ^168^ ^]^ They found that a fascinating interplay where benzylamine (BnAm) and FA^+^ underwent a transformative reaction generated new substances, i.e. ─NH_3_ and BnFA^+^. ─NH_3_ could bind to Pb^2+^ ions, optimize crystallization processes, and modify structural defects. While, BnFA^+^ was speculated to have two possible combination modes with 3D perovskites, highly related to the adding concentration (Figure 4d): one was to selectively passivate the A‐site vacancies on the capped surfaces of 3D crystals; the other was to form 2D perovskite phases surrounding 3D crystal domains. At an optimal doping concentration, the encapsulated device achieved an impressive *T*_80_ stability of 2460 h under extremely accelerated aging conditions.

#### 3.1.3 Solvent Engineering

**Figure 5:**
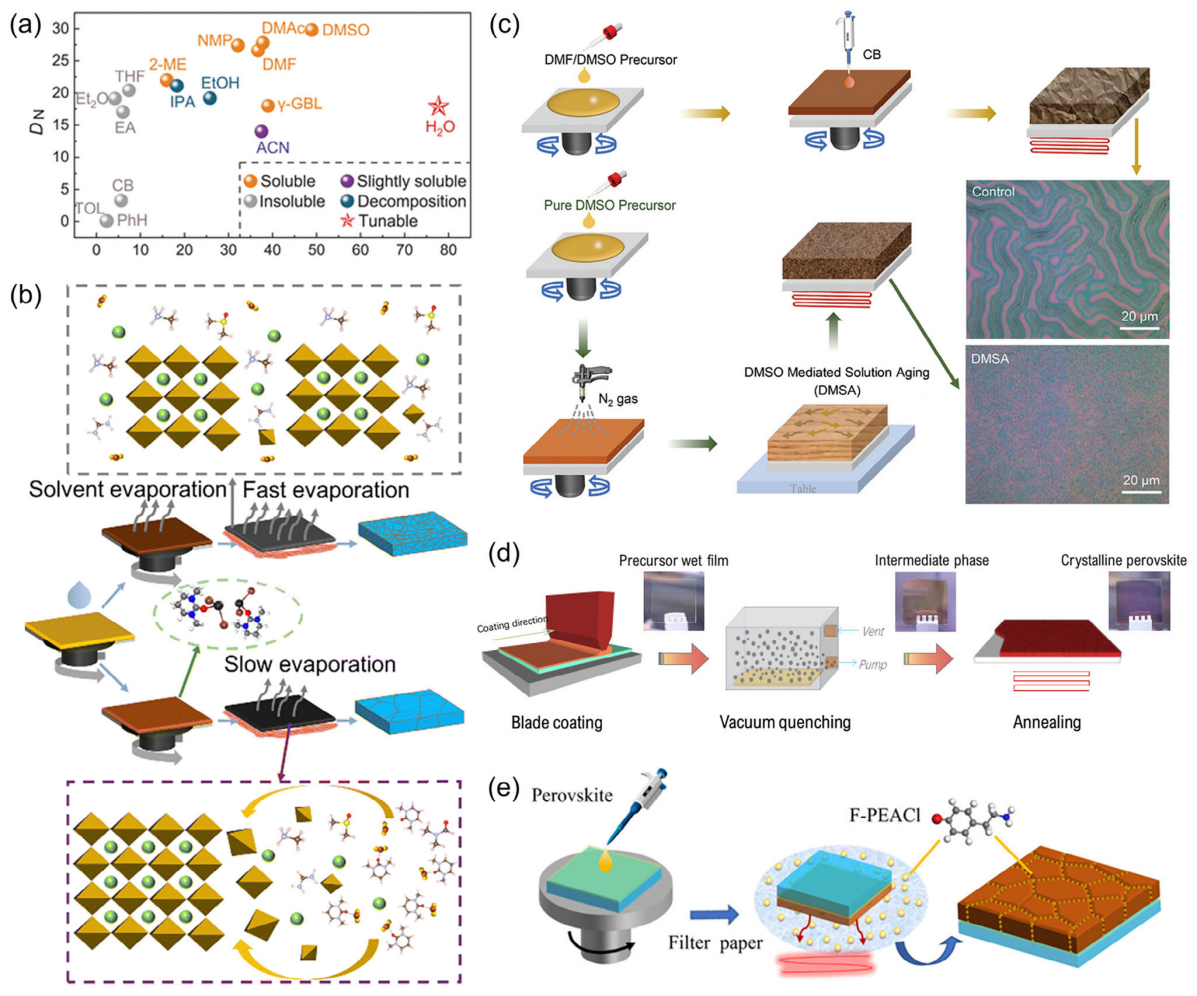
a) D_N_ and ɛ_r_ of the various solvents. Reproduced with permission.^[^ ^182^ ^]^ Copyright 2024, The American Association for the Advancement of Science. b) Schematic illustration of the growth pathways pf the perovskite film with DMPU. Reproduced with permission.^[^ ^40^ ^]^ Copyright 2024, The Royal Society of Chemistry. c) Preparation procedures and top‐view optical microscopic images of perovskite films from DMF/DMSO precursor ink through antisolvent quenching and from pure DMSO solution through gas quenching and DMSA treatment. Reproduced with permission.^[^ ^183^ ^]^ Copyright 2024, Wiley‐VCH GmbH. d) Schematic diagram of vacuum‐assisted blade coating for deposition of perovskite film. Reproduced with permission.^[^ ^184^ ^]^ Copyright 2023, American Chemical Society. e) Schematic of normal annealing and passivator‐assisted annealing processes. Reproduced with permission.^[^ ^185^ ^]^ Copyright 2024, Wiley‐VCH GmbH.

The selection of solvents for fabricating perovskite films is of well‐established importance for manipulating the crystallization kinetics to attain high‐quality perovskite films. The correlation between Gutmann donor number (D_N_) and dielectric constant (ɛ_r_) provides insights into the solubility capabilities of solvents for both organic and inorganic halides (**Figure** **5**a). According to this principle, solvents can be generally divided into precursor solvents that can dissolve perovskites, antisolvents that are insoluble to perovskites, decomposition solvents that only dissolve organic halides and dissolution‐variable solvents (e.g., H_2_O). The benign solvents that well dissolve perovskites commonly exhibit Lewis basicity because of the presence of oxygen‐containing double bonds (denoted as OXRs).^[^ ^169^ ^]^ An additional set of two parameters, D_N_ and Kamlet Taft (β), can further indicate the competitive interactions between perovskites and these solvent molecules, which might generate two possible intermediate structures in solution: Pb^2+^‐OXR complexes via coordination bonds and (A^+^⋯OXR) complexes via hydrogen bonds. DMF is widely employed in dissolving perovskites, however, its limited solubility to inorganic halides requires adding other solvents with stronger D_N_, such as DMSO.^[^ ^170^, ^171^ ^]^ Although DMSO, with higher boiling temperature, can to some extent retard crystallization rate and promote chemical homogeneity, the DMF/DMSO solvent system still encounters several challenges. For example, a comparably narrow processing window, susceptibility to moisture and oxygen (rendering perovskite spoiled), weak complexation of PbI_2_ (leaving residues behind), and toxicity (restricted for use in glove boxes).^[^ ^171^, ^172^ ^]^ Wang et al. tailored triple mixed solvents by partially substituting DMSO with DMPU, which has a larger D_N_ and stronger coordination with PbI_2_, successfully extending the antisolvent dropping window and slowing down the perovskite transformation process (Figure 5b).^[^ ^40^ ^]^ DMPU was found to completely convert PbI_2_ into perovskites, make the interface energy levels between hole transport and perovskite layers more aligned, and reduce the density of defects. As a result, the champion 1.64 eV‐device yielded a PCE of 21.9%, and a *V*_oc_ of 1.256 V, and outstanding ambient and light stabilities. Distinct from DMF and DMSO, NMP‐induced crystallization behavior involves more stable double intermediate adducts of FAI∙NMP and PbI_2_∙NMP, which are more conducive to the formation of α‐phase perovskites in both antisolvent‐assisted molecular dynamics and thermodynamics, thereby effectively improving defect ecosystem.^[^ ^169^, ^173^, ^174^, ^175^ ^]^ Tang et al. blade‐coated dense and uniform FA_0.9_Cs_0.1_Pb(I_0.6_Br_0.4_)_3_ films from NMP‐single‐solvent, combined with 2D/3D surface reconstruction by F‐PEAI, achieving a PCE of 18.92% with a *V*_oc_ of 1.26 V, owing to both passivated bulky and surface non‐radiative recombination centers.^[^ ^175^ ^]^ Zhang et al. studied the effect of EA and alcohol‐based antisolvent mixtures on the perovskite phase transition in DMF/NMP system.^[^ ^174^ ^]^ They discovered that minor IPA governed the crystallization process by restraining iodine ion migration. The optimized perovskite film exhibited a nearly eightfold increase in the photoluminescence quantum yield, suggesting a significant reduction in bulk non‐radiative recombination and enhanced QFLS. Li et al. conducted a comparative study on (111) and (001)‐oriented WBG perovskite films using anti‐solvent engineering.^[^ ^176^ ^]^ They observed that, although both orientations exhibited similar photovoltaic performance, (111)‐oriented devices demonstrated superior photo/voltage stability due to their ability to hinder ion migration pathways along the electric field direction.

For the sake of environmental friendliness and health & safety, along with industrial requirements for ambient and scalable fabrication, green solvents are receiving much attention. Wang et al. proved the feasibility of using pure ionic liquid MAAc to prepare CsPbI_3‐x_Br_x_ devices in air.^[^ ^171^ ^]^ Ac‐based solvent systems form more stable bidentate complexes with Pb^2+^ through chelation (O⋯Pb), avoiding the destruction of water molecules.^[^ ^171^, ^177^, ^178^ ^]^ The stronger hydrogen bonding between halogen and hydrogen atoms protects the iodine ions from oxidation. Therefore, these features ensure high‐quality perovskite films with fewer grain boundaries, high flatness, and no pinholes. Also, Ac^−^ is viewed as one of pseudo halogens, which can passivate halide vacancies to diminish defect density, suppress ion migration, and enhance stability.^[^ ^179^ ^]^ Tan's team recently reported a two‐step sequential deposition technique that could be applicable in ambient atmospheres and for large scales, during which green alcohols were used to dissolve organic salts and react with as‐deposited lead halides.^[^ ^180^ ^]^ They elucidated that the compatibility of alcohols with water molecules and their impact on perovskite growth kinetics were closely related to their carbon chain length, which in turn influenced their saturation vapor pressure and polarity. The optimal nBA‐treated single junction WBG devices delivered 20.8% PCE for a photoactive material of 1.68 eV. In their recent work, a ternary green solvent mixture of DMSO/ACN/EtOH has been demonstrated to possess suitable DN and AN (coordinating complexes between Pb^2+^, I^−^, Br^−^, and solvent molecules), moderate vapor pressure (controlling solvent evaporation), and anti‐protonation properties (preventing degradation of perovskite ink).^[^ ^181^ ^]^ This strategy enabled the reproducible fabrication of large‐area blade‐coated WBG PSCs with high efficiency in an ambient of 40% RH.

Water can also strikingly serve as a solvent for perovskite synthesis. However, it needs to be paired with halogen acid to improve its solubility for lead halides, and assures sufficient purity to avoid the impurity ions to form traps. Zhu et al. synthesized commercial‐grade high‐purity perovskite microcrystals in an aqueous solution, including components with wide bandgaps (e.g., 1.73 eV‐FA_0.8_Cs_0.2_Pb(I_0.7_Br_0.3_)_3_).^[^ ^182^ ^]^ Notably, devices prepared by re‐dissolving perovskite crystals as precursors exhibited remarkably improved PCE (20%) and *V*_oc_ (1.27 V). This is because the precursor derived from crystal re‐dissolution retains the inherent excellent properties of crystals, such as precise stoichiometry, low trap density, high crystallinity, and highly‐ordered intermediate clusters, rendering resultant perovskite films with high qualities. Therefore, this technology will exert a substantial positive impact on the perovskite industry, not only tremendously reducing the manufacturing costs of high‐quality precursor materials in a more environmentally friendly way, but potentially advancing the efficiencies of solar cells within the field.

#### 3.1.4 Fabrication Techniques

The conventional anti‐solvent spin‐coating technique allows for a vast majority of polar solvents extracted out of the samples to be prepared in an instant, rapidly bringing about massive heteronuclei and intermediate perovskite phases. Subsequent thermal annealing stimulates anisotropic growth and generates numerous grain boundaries. Given the existence of bromine and cesium constituents in WBG perovskites, their swift crystallization nature further accelerates the structural and energetic disorders in systems, culminating in spatial element inhomogeneity. Moreover, anti‐solvent solution processing adopts a bottom‐up crystal growth mode which may generate voids at the bottom due to solvent evaporation and intensify surface roughness and defect density (e.g. typical surface “wrinkles”). From an application perspective, this approach also constrains its use solely in the fabrication of small‐area prototypes for laboratory research, as large‐area spin‐coating can result in film thickness discrepancies from the center to the periphery, as well as worse defect states. Therefore, such a dilemma calls for alternative preparation techniques to regulate crystallization dynamics, homogenize components, and be applicable for large‐size production of WBG perovskite films.

Gas quenching takes advantage of a stream of gas (such as N_2_, and Ar) to remove solvent slowly.^[^ ^42^ ^]^ It first induces the appearance of nucleation‐prone phases (e.g., Br‐rich phase) at shallow surfaces in a much milder manner, and then foster the growth of inner perovskite along a longitudinal gradient. Concurrently, at the horizontal level, depending on solvent volatilization, gas quenching can greatly decelerate the rate of intermediate phase transition in perovskite, thus effectively relieving film stress prior to annealing and eliminating surface “wrinkles”.^[^ ^186^, ^187^ ^]^ WBG perovskite films produced by this method generally exhibit larger grain sizes, longer carrier diffusion lengths and lifetimes, lower defect densities, and markedly improved halide phase stability. Lian et al. suggested an additional static aging process following gas quenching for systems using pure DMSO solvent, because the high viscosity of DMSO requires a buffer period to rectify uneven films across the surface tension gradients.^[^ ^183^ ^]^ Compared to the samples prepared by anti‐solvent spin‐coating method, the target perovskite films exhibited strain‐released, flat, and smooth apparent morphology (Figure 5c), indicating a low density of surface defects and suppressed non‐radiative recombination.

Vacuum quenching or gas pumping is another dry method employed to remove excess coordinating solvents from pre‐formed wet films, achieving supersaturation and initiating nucleation (Figure 5d).^[^ ^154^, ^175^, ^184^ ^]^ This process significantly extends the transformation stage of the intermediate phase of perovskite to the minute domain, thereby moderating the crystallization kinetics and avoiding the residence of untransformed lead halides and their heterogeneous phases. It is worth noting that parameters such as vacuum/pumping time, vacuum level, and solvent vapor pressure have a crucial impact on the final film quality. This technique is particularly well‐suited for the fabrication of large‐area perovskite modules. For instance, Gao et al. reported an efficiency of 17.63% for a 19.34 cm^2^ 1.77 eV‐perovskite module via gas‐pumping onto as‐spin‐coated perovskite films for 5 s.^[^ ^154^ ^]^

Apart from controlling the nucleation process, crystalline growth during annealing can be slackened through repeated cycles of dissolution and crystallization caused by either residual or intentionally introduced solvent. Wang et al. described a side‐down close‐space annealing method in which freshly cast perovskite samples were flipped onto a filter paper on a hotplate.^[^ ^188^ ^]^ Unlike typical direct‐exposure thermal annealing, the remaining DMSO in wet films escaped the confined space more slowly, which promoted Ostwald ripening and oriented attachment of perovskite crystals. It melted grain boundaries, increased grain domains, prevented voids, and enhanced charge‐carrier properties. 1.75 eV‐WBG devices achieved boosted efficiency and *V*_oc_ from 17.22% and 1.22V to 18.58% and 1.226 V, respectively. In a recent advancement of this methodology, Zhao et al. incorporated volatile F‐PEACl which was dissolved in IPA solution onto filter paper.^[^ ^185^ ^]^ This allowed F‐PEA^+^ for efficient penetration of both the surface and bulk of materials for the purpose of defect passivation (Figure 5e), ultimately achieving impressive 21.28% and 20.24% PCE for 1.68 eV and 1.73 eV solar cells, respectively.

It is worth mentioning that Zheng et al. recently published a pioneering study detailing an innovative wet post‐polishing technique to reconstruct ultra‐smooth and enhanced crystalline perovskite surfaces.^[^ ^189^ ^]^ Through the application of AlO_x_ nanoparticles, the superficial layer that was rich in defects and lattice strain underwent polishing and then exposed Br‐rich crystal facets due to stronger hardness. The resulting surface effectively suppressed interface charge carrier losses and ion migration, and established favorable energy band alignments. PSCs with 1.67 eV E_g_ set a groundbreaking PCE of 24.48% (certificated 23.67%) and preserved 80% performance at MPP operation for 1505 h. This work will guide the application and transformation of this technique in the fabrication of high‐performance WBG PSCs.

### 3.2 Charge Transport Layers

CTLs are key components of PSCs with the essential function of selectively extracting majority carriers into electrodes from absorbers while adequately blocking minority carriers. A negligible offset in energy bands between perovskites and charge transport materials is ideally advantageous, as it prevents carrier accumulation, depletion, or inversion. It is imperative that CTLs exhibit high carrier mobility and conductivity for the target carriers, alongside a low density of bulk and surface defect states, so as to cast off the non‐radiative recombination.^[^ ^34^ ^]^ Nevertheless, prevalent charge transport materials encounter serious challenges. The maximum valence band levels of hole‐transport layers (HTLs) are grossly mismatched with the much deeper ones of WBG PSCs,^[^ ^190^ ^]^ leading to a pronounced band drop‐off that heightens the potential Schottky barriers, impedes charge‐carrier transfer and transport, and exponentially increase trap‐mediated recombination possibilities at interfaces.^[^ ^34^ ^]^ Metal oxide CTLs, despite their seemingly better transparency and conductivity, are often rich in surface defects. For instance, uncoordinated Ni^≥3+^ cations on NiO_x_ surface can induce localized regions enriched with PbI_2‐x_Br_x_, one type of hole traps, decreasing the selectivity and collection efficiency of holes.^[^ ^62^, ^191^, ^192^ ^]^ Organic polymer CTLs suffer from significant optical parasitic absorption (cause photocurrent loss),^[^ ^193^, ^194^ ^]^ hydrophobicity (affect the perovskite film quality if coated onto buried CTLs),^[^ ^195^, ^196^ ^]^ and low carrier mobility (slow carrier extraction rate down).^[^ ^197^, ^198^ ^]^ In addition, metal oxide CTLs, alongside some certain acidic and hygroscopic organic materials (e.g., PEDOT:PSS) undergo acid‐base neutralization and redox reactions with perovskites, accelerating the irreversible degradation of materials and the formation of irreparable defective areas. Therefore, the optimization of existing CTL material systems and the development of novel transport layer materials tailored for WBG perovskites (more critical) hold strategic significance.

Huang et al. added choline chloride to SnO_x_ to coordinate Sn^2+^ and non‐coordinated Pb^2+^ on the contact surface, promoting efficient charge extraction at the interface and increasing the *V*_oc_ of CsPbI_2_Br upright devices from 1.2 V to 1.315 V.^[^ ^199^ ^]^ Moreover, Jiang et al. synthesized Mg and Cl co‐doped ZnO to promote the vertical orientation growth of CsPbI_2_Br perovskite on the substrate.^[^ ^199^ ^]^ This approach reduced leakage current, resulted in a *V*_oc_ surpassing 1.3 V, and a consistently outputting efficiency exceeding 17%. Fan et al. addressed the hygroscopicity and Li^+^ ion migration challenges of Spiro‐OMeTAD by adding 1,2‐Bis(perfluoropyridin‐4‐yl)disulfane (BPFPDS) which is rich in highly electronegative ions.^[^ ^198^ ^]^ This strategy not only achieved a remarkable *V*_oc_ of 1.29 V and an impressive PCE of 21.95% for all‐inorganic CsPbI_3_ PSCs, but demonstrated exceptional stability in air‐storage and at MPP operation for 3000 h and 1200 h, respectively. In a p‐i‐n device configuration, Sun et al. reported a hybrid fullerene ink (C_60_:PCBM:ICBA) that exhibited higher electron mobility compared to individual components.^[^ ^197^ ^]^ Mixed fullerenes constructed a type II band alignment with perovskite and passivated interfaces with their branches, beneficial to the efficient collection of photogenerated carriers.

**Figure 6:**
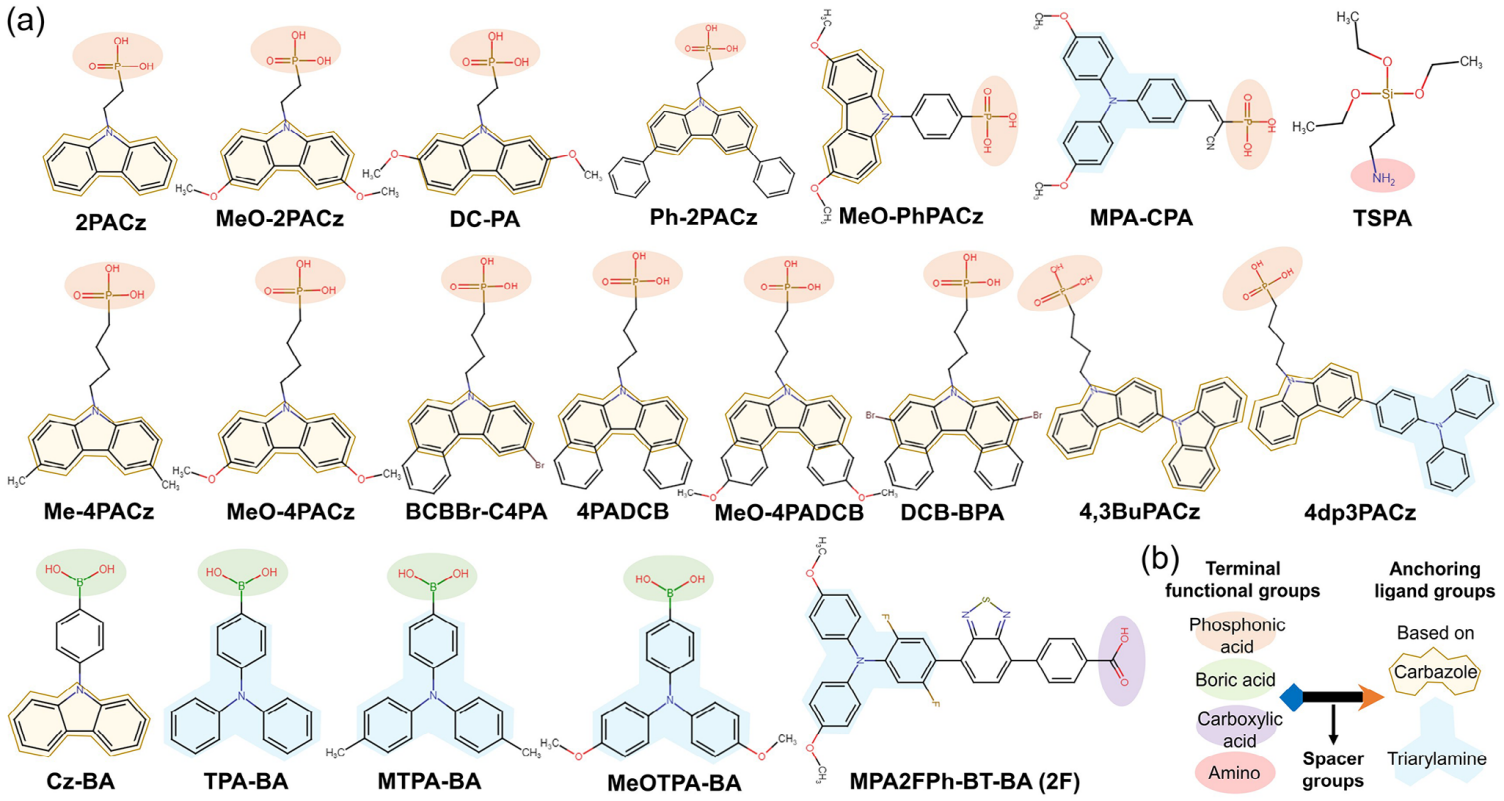
a) Structural illustration of SAM molecules reported in WBG PSCs with distinct color patterns grouping the same moieties. b) Schematic overview of SAM structures with summarized moiety groups.

On the other hand, self‐assembled monolayer (SAM) molecules, particularly hole‐selective materials (see **Figure** **6**a), have contributed a lot to the cutting‐edge performance of WBG PSCs in a p‐i‐n device configuration. This is credited to the electric dipoles, generated by the electronegativity contrast at the molecule ends and characteristic of the molecular dipole moment, that shift the apparent Fermi level of the contacting substrate, align energy levels, suppress interfacial non‐radiative recombination, enhance internal built‐in electric fields and improve the overall QFLS status.^[^ ^200^, ^201^ ^]^ This benefit is closely related to the magnitude of the dipole moment and the direction of action.^[^ ^200^, ^202^ ^]^ Figure 6b depicts three components of a SAM molecule: an anchoring ligand group, a terminal functional group, and a spacer moiety that bridges them. The terminal groups are primarily based on functional derivatives of carbazole and triarylamine, which serve as electron donors and selectively receive photogenerated holes from the perovskite layers. However, the interaction between the carbazole moiety and the perovskite is limited, resulting in insufficient suppression of interface defects. Although the introduction of methyl groups can passivate perovskites, the higher carbon‐hydrogen bond density makes it hydrophobic to the perovskite precursor solution, leading to the formation of pinholes and buried voids. Alternatives, such as methoxy or halogen groups, can be considered. It should be noted that the introduction of different groups leads to different dipole moments of the entire molecule, which depends not only on the electronegativity of the groups themselves but also on their positions and symmetries.^[^ ^203^, ^204^ ^]^ This is primarily because different substitution positions alter the electron‐donating ability at the terminal end. Furthermore, undesired SAM aggregation may occur due to the carbazole moiety through π‐π interactions, which both impact the solubility and uniform deposition of SAM molecules. Hence, designing a unique non‐coplanar helical structure of the 7H‐dibenzo[c,g]carbazole backbone (4PADCB, MeO‐4PADCB, DCB‐BPA, DCB‐Br‐2) can enhance the steric repulsion of the end groups and prevent molecular stacking.^[^ ^205^, ^206^, ^207^, ^208^ ^]^ Additionally, this structure can weaken the influence of methoxy groups on the dipole moment and make the molecular skeleton parallel to the substrate, which is more conducive to charge transfer. Introducing a phenyl ring at the relative end of the carbazole moiety (Ph‐2PACz) can also weaken the π‐π interactions by increasing the intermolecular distance.^[^ ^209^ ^]^ Another strategy is to design asymmetric functional groups (BCBBr‐C4PA, 4,3BuPACz, 4dp3PACz) to achieve a stronger electric dipole moment and improved wettability.^[^ ^210^, ^211^, ^212^ ^]^ Furthermore, the carbazole moiety has a low delocalized electron density, limited charge transfer ability, and is prone to photodecomposition under illumination. Replacing it with a stronger electron‐donating molecule such as triphenylamine can strengthen its interaction with the perovskite,^[^ ^211^, ^213^ ^]^ increase the molecular dipole moment, minimize defect density and optimize the extraction of hole charge carriers. Triphenylamine with a propeller‐like structure also aids in the molecule dispersion in solvents.^[^ ^214^ ^]^ Zhu et al. designed a multifunctional MPA2FPh‐BT‐BA (2F) molecule by fluorinating one benzene ring of triphenylamine, and concurrently introducing a benzothiadiazole intermediate group.^[^ ^215^ ^]^ This SAM enabled more effective passivation and repair of non‐coordinated perovskite ions and oriented their crystalline growth. Overall, by optimizing the end functional groups, the performance of WBG PSCs is significantly enhanced, and the *V*_oc_ can reach 80% or even approach 90% of S‐Q limits.

The connecting spacers can be non‐conjugated alkyl chains or conjugated aromatic groups.^[^ ^214^ ^]^ The former, influenced by the weakened van der Waals interactions because of longer chain length, results in electron decoupling effects that limit charge transport efficiency. A sluggish charge transfer rate may induce hole accumulation near interfaces,^[^ ^163^, ^216^ ^]^ thereby lowering the activation energy for ion migration and promoting I^−^‐movement towards grain boundaries, and then triggering phase segregation. In contrast, the latter allows for longitudinal charge transfer thanks to changed electron delocalization, facilitating carrier transportation.

The anchoring groups, as electron acceptors within the SAM molecules, bind to secondary CTLs or transparent conductive oxide (TCO) electrodes through the chemical bonds that are also regarded as the final channels for carrier transfer. The transfer efficiency is influenced by variables like contact resistance and work function differences at interfaces.^[^ ^217^ ^]^ The widely employed anchoring groups in WBG perovskite cells include phosphonic acid (─PO(OH)_2_), carboxylic acid (─COOH), and boric acid (─B(OH)_2_). They can engage in either condensation reactions with hydroxyl groups on the surface of metal oxides (MO_x_) and passivate oxygen vacancies, forming covalent P─O─M bonds, or coordinate covalent P═O─M bonds with metal atoms at the interface.^[^ ^218^, ^219^, ^220^ ^]^ The anchoring mechanism of other groups is analogous to this. In light of the crucial role of these bonds in providing robust charge transport channels, it is of utmost importance to ensure strong ─OH adhesion on the substrate surface and a higher metal atom exposure density, which, in turn, enhances uniform and dense SAM packing and strong adhesion. However, previous investigations have revealed the local accumulation and easy detachment of SAM molecules caused by the roughness and fragile adsorbed hydroxyl groups of substrate surfaces.^[^ ^218^, ^221^ ^]^ To address such issues, the introduction of additional metal oxide layers (such as NiO_x_, IZrO, ITO nanocrystals) or surface treatments (such as acidification and UV ozone treatment) can enhance reliably bonded hydroxyls, increasing the coverage and uniformity of SAM molecules.^[^ ^59^, ^205^, ^218^, ^221^, ^222^ ^]^ Furthermore, phosphonic acid groups can also anchor onto materials like PEDOT:PSS or PTAA through interactions with sulfur atoms on the thiophene rings or by acid‐base reactions with nitrogen atoms possessing basic lone pairs, respectively.^[^ ^223^, ^224^ ^]^ This anchoring mechanism implies the wide applicability of SAMs on various substrates. Whereas, the strong acidity of phosphonic and carboxylic acids may potentially corrode MO_x_ or TCO surfaces and accelerate hydroxyl proton dissociation. To overcome this issue, Guo et al. synthesized SAM materials based on the weaker acidity of boronic acid anchors.^[^ ^210^ ^]^ Devices based on boronic acid SAMs demonstrated superior operational stability compared to those with phosphonic acid groups in ISOS‐D‐1 and ISOS‐L‐1 stability tests. Mann et al. reported a SAM based on 3‐(Triethoxysilyl)propylamine (TSPA) that utilized amino (─NH_2_) hydrogen bonding to anchor the NiO_x_ layer.^[^ ^213^ ^]^ The differential electronegativity between the amino group and the silane group in TSPA formed a favorable dipole, which compensated for the energy level discrepancy and boosted the built‐in electric field. The modified devices finally exhibited notably improved photo‐voltage and ‐current, achieving an efficiency of 20.21% for a 1.7 eV WBG device.

The utilization of mixed‐SAMs or co‐SAMs has been reported to improve performance. It can be achieved by blending different as‐developed SAMs at ratios or sequentially depositing them,^[^ ^146^, ^225^ ^]^ or by cooperating with some linear SAM small molecules (such as 6dPA,^[^ ^226^ ^]^ IAHA^[^ ^204^ ^]^). This benefits to form a denser and more uniform SAM layer, improving the wettability of the SAM layer on the perovskite, optimizing the interface energy levels for better alignment, and reducing interfacial defect density. Moreover, the addition of small‐sized molecules not only fills the gaps between anchoring groups but also passivate surface defects of the perovskite with exposed functional groups. In addition to its predominant use in the inverted structure, there have been exploratory attempts to utilize SAMs in the n‐i‐p‐type devices.^[^ ^227^ ^]^ Note that electron‐selective contact SAMs have also gained attention and are being developed.^[^ ^217^, ^228^, ^229^ ^]^ A few works have preliminarily explored the viability of fullerene‐derived SAMs on n‐i‐p WBG devices, demonstrating their substantial advantages over immobilizing interfacial iodide ions and superior charge extraction.^[^ ^230^, ^231^, ^232^ ^]^

### 3.3 Interface Passivation

In light of the dominant role of interface nonradiative recombination in QFLS losses (see Section 2.2), interface passivation engineering plays a critical role in reducing surface defects of both perovskite and CTLs, alongside providing a smooth pathway for charge carriers to be extracted and transported. To this end, the modification interlayers are required to possess the capability of strongly interacting with the contact substances and regulating the energetic alignment. In addition, if employed as buried interface modifiers, they should enhance the compatibility between perovskites and substrates, and strengthen the crystalline growth of perovskites. So far, successful implementations have involved introducing low‐dimensional (0D, 1D, and 2D)/three‐dimensional (3D) surface heterostructures, multifunctional ionic liquid modifications, small molecules or polymers as passivating agents, amines and excess organic cation halides for surface optimization. These strategies additionally prevent adverse photochemical reactions between perovskites and transport layers to a certain extent, significantly maintaining the operational stability of devices.

#### 3.3.1 HTL/Perovskite

Conjugated poly[(9,9‐bis(30‐((*N*,*N*‐dimethyl)‐*N*‐ethylammonium)‐propyl)‐2,7‐fluorene)‐alt‐2,7‐(9,9‐dioctylfluorene)] dibromide (PFN‐Br or PFN‐P_2_) polymer commonly serves as the interface compatibilizer to functionalize the hydrophobic PTAA or Poly‐TPD hole transport layers, ensuring a high‐quality deposition of photoactive layers in an inverted device structure.^[^ ^27^, ^31^ ^]^ More importantly, PFN‐Br has been proven to principally diminish trap‐assisted recombination at interfaces, in turn granting minor QFLS loss between perovskite and HTL layers, resembling the performance of a neat perovskite film.^[^ ^34^, ^233^ ^]^ To replace PFN‐Br, Gaprioglio et al. evaluated the feasibility of a series of cation‐anion pairs on PTAA, involving mobile small ions (Li^+^, Br^−^) and immobile large ions (TMA^+^, TEA^+^, TFSI^−^).^[^ ^234^ ^]^ They concluded that TEA‐TFSI with both large but immobile ionic characteristics performed the best interfacial passivation effect, upgrading all photovoltaic parameters.

Regarding surface modifications on NiO_x_ layers, cross‐linkable p‐type polymer VNPB,^[^ ^235^, ^236^, ^237^ ^]^ organometallic dye molecule N719,^[^ ^62^, ^238^ ^]^ and donor–acceptor‐pair‐contained small molecules (IQTPA and IQTPAFlu)^[^ ^239^ ^]^ have been explored over past years. VNPB can enhance the wettability of perovskite precursor on NiO_x_, allowing the more uniform and pinhole‐free photoactive layers. Its faster hole extraction rate and higher formation energy barriers to ion‐vacancy defects can result in an average increase of 30–50 mV in *V*_oc_.^[^ ^236^, ^237^ ^]^ N719 encompasses high‐density electron‐donating groups such as carboxylic acid, thiocyanate, and carboxylic ester, enabling strong intermolecular interactions with the electron‐deficient regions of both perovskite and NiO_x_.^[^ ^62^ ^]^ This effectively mitigates defect formation at the dual interfaces. Additionally, the transparency of NiO_x_ in the visible light region is refined due to the reduced Ni^3+^ point defects by N719, thereby alleviating optical losses in the device. Benefiting from the difference in electron density between indole and quinoxaline units, IQTPA and IQTPAFlu interlayers offer positive dipole moments to increase the built‐in electric field, which improves the energy band alignment and facilitates interface charge extraction.^[^ ^239^ ^]^ At the same time, they possess multiple Lewis basic nitrogen sites that can strongly interact with uncoordinated Pb^2+^ in perovskites via lone‐pair electrons, curbing the formation of defects. Apart from these, buried interface passivation by ammonium salts is also a common solution. Recently, Zhang et al. implemented a ligand anchoring heterointerface strategy via modifying NiO_x_/PTAA/Al_2_O_3_ NPs substrates with 3‐MeOPEACl.^[^ ^92^ ^]^ The methoxy groups bonded with Al_2_O_3_ through a chemical reaction, while ammonium terminals passivated perovskite undersurfaces by assisting in vertical crystal growth and homogenizing halide distribution. The champion 1.71 eV WBG device yielded a PCE of 20.83%, alongside extraordinary shelf stability over 7900 h.

In the realm of n–i–p structured devices, the passivation of the perovskite/HTL interface is preferentially directed towards mitigating the various defects present on the upper surface of the perovskite layer. These defects encompass electron‐deficient species, such as uncoordinated Pb^2+^ ions, halide vacancies, and Pb° clusters, as well as electron‐rich defects including dissociative halide ions, anti‐site PbI_3_^−^ anions, and cation vacancies. Equally crucial, the passivation contributes to optimizing the energy levels by creating gradients at interfaces and manipulating the charge transfer processes that foster exceptional hole collection performance. For instance, a zwitterionic carbamoyl‐guanidine amidino urea salt (CuanCl) was post‐treated on perovskite surface, of which C═O bonds coordinated with Pb^2+^ defects, ═NH─ cations interacted with I^−^ and FA vacancies, and Cl^−^ ions occupied halide vacancies, resulting in a significant decrease in shallow surface defect density.^[^ ^240^ ^]^ Ye et al. sequentially deposited FAI and TOPO modifiers to produce an in situ surface heterojunction and a passivation effect on Pb^2+^ cations, collaboratively enhancing optical absorption and reducing interfacial charge recombination.^[^ ^125^ ^]^ Additionally, Mussakhanuly et al. adopted FABr:2PACz mixtures to eliminate under‐reacted PbI_2_ and Pb^0^ residues.^[^ ^227^ ^]^ The SAM also kept photogenerated carriers from funneling into I‐rich clusters, thus retarding halide segregation. Overall speaking, these strategies shared a commonality in inducing an ultrathin layer with a valence band maximum (VBM) positioned between the VBM of perovskite and the highest occupied molecular orbital (HOMO) level of Spiro‐OMeTAD (used in these works), which favorably bent the contact bands and promoted hole transport. As a result, optimized WBG devices exhibited a universal improvement in *V*_oc_ by 20–80 mV, FF reaching ≈80%, and a remarkable increase in efficiency. Even more impressive is their long‐term stability over thousands of hours, attributed to the enhanced resistance against light, heat, moisture, and oxygen due to reduced defects.

#### 3.3.2 Perovskite/ETL

Organic halide salts, as universal surface passivation, have been extensively applied in the post‐treatment modification of inverted WBG perovskite devices. Numerous investigations have positively demonstrated their effectiveness in markedly diminishing the density of defect states at the perovskite/ETL interface, releasing surface strain, aligning interfacial energy levels, mitigating non‐radiative recombination losses, improving open circuit voltage, and strengthening the stability of devices against moisture and oxygen ingress. Within the organic part, alkyl chain‐based organic cationic ligands represent a prevalent design strategy. Tailoring these ligands with customized functional groups (e.g., ─NH_2_, ─NH_3_, ─CH_3_, benzene rings, and their fluorinated, nitrided, sulfided, hydroxylated, and carboxylated derivatives, etc.), at one or both ends, enables robust interactions with perovskites. This involves either anchoring the organic molecules at defect sites atop the perovskite layers, or transforming the shallow layer or the residual PbI_2_ substances into a low‐dimensional perovskite‐like layer. Pu et al. adopted a dual 1,3‐propane‐diammonium iodide (PDADI):formamidinium iodide (FAI) approach to alleviate surface strain on 1.77 eV perovskite films, as a result of elevating the formation energy of halide vacancies.^[^ ^241^ ^]^ The ─NH_2_ functional group in PDADI passivated uncoordinated Pb^2+^ and immobilized halide ions via hydrogen bonds. Under continuous 1‐sun light‐soaking, the optimized film immersed in a toluene solution exhibited no I_2_ absorption peak resulting from ion migration degradation, confirming the enhanced photostability. Through chemical surface polishing, bis‐ammonium alkyl ligands featuring two ─NH_3_ groups (e.g., EAD^2+^, PDA^2+^, etc.) can increase apparent grain sizes, and fill A‐site and X‐site ion vacancies at surfaces and grain boundaries. More inspiring, they are interpreted to repel minority carriers via field effect to extend carrier lifespan, thus suppressing tail‐states‐induced non‐radiative recombination.^[^ ^242^, ^243^ ^]^ In addition, this class of materials is also believed to establish a conducive n‐type 2D perovskite layer at interface, offering a gradient energy level for electron transport to the ETL smoothly.^[^ ^244^ ^]^ It is particularly noteworthy that the passivation effect of diammonium salts is closely associated with the alkyl chain length inside, of which essence is the differentiated electrostatic binding force and the affected carrier conductivity.^[^ ^243^, ^245^ ^]^

Phenethylammonium (PEA^+^) moiety based on benzene ring and ─NH_3_ can facilitate the formation of a stable Ruddlesden‐Popper (RP) phase on the perovskite surface. The π‐π interaction between its aromatic rings reinforces the structural rigidity of the organic spacer layer, curbing the thermal motion of halogen atoms in the connected inorganic layer, and reducing electron‐phonon coupling, which in turn enhances the carrier mobility.^[^ ^246^, ^247^ ^]^ However, in this case, due to the face‐to‐edge stacking mode,^[^ ^248^ ^]^ the terminal ammonium part would be offset, leading to staggered adjacent inorganic layers, which hampers interlayer charge transfer. To this end, one solution is to boost the intramolecular interaction by introducing functional groups or doping heteroatom. They are capable to adjust the interlayer spacing and the symmetry of the out‐of‐plane direction of the inorganic layer. For example, F‐PEA^+^ was discovered to connect by means of face‐to‐face benzene rings stacking, aligning the terminal ammoniums and the perovskite octahedron almost vertically. This configuration promoted interlayer electronic coupling and carrier transport.^[^ ^248^ ^]^ Furthermore, Zhou et al. found that the introduction of highly electronegative F atoms enhanced the dipole moment and molecular polarity of organic cations, fostering intramolecular charge transfer. Compared with bare PEAI and HO‐PEAI, the 2D/3D heterojunction structure formed by F‐PEAI demonstrated superior halogen phase stabilization, thanks to the significant improvement in ion activation energy. The champion device based on 1.78eV perovskite achieved a record‐breaking *V*_oc_ of 1.35 V and retained 92% efficiency after 500 hours of illumination.^[^ ^249^ ^]^ In parallel, Jiang et al. theoretically unveiled that the formation of dual‐ion EAD_Pb+I_ Schottky defects would induce n‐type electrical doping.^[^ ^250^ ^]^ Therefore, they combined EDAI_2_ and 3‐fluorophenethylamonium iodide to fabricate an n‐doped low‐dimensional heterojunction layer that cleared up the interface barrier for carrier extraction. As a consequence, they culminated in a cutting‐edge 1.34 V *V*_oc_ and 20.37% PCE for 1.77 eV devices. Promptly, Wang et. al. recently underscored the significance of a 4‐fluorophenethylamine‐based 2D perovskite ultrathin layer in mitigating contact losses and heterogeneities of thermally evaporated C_60_ onto the perovskite surface.^[^ ^45^ ^]^ Through the co‐utilization of 4‐trifluoromethyl‐phenylammonium (CF3‐PA), endowed with greater charge extraction and transfer capabilities, they set a new pinnacle in performance metrics with the groundbreaking 1.35 V *V*_oc_ and 20.5% PCE in the same device system. Conversely, Huo et al. pursued an alternative approach by deprotonating a portion of PEA^+^ via an annealing process to generate volatile PEA^0^.^[^ ^251^ ^]^ The remaining PEA^+^ spontaneously formed a dipole layer instead of a conventional 2D phase. It not only passivated surface defects, but is better to align the conduction band energy level of perovskite with that of C_60_. As a result, this improvement delivered a high *V*_oc_ of 1.26 V, with only 0.41 V loss, and an impressive PCE of 20.61%, for 1.67 eV WBG solar cells.

Other organic salts are equipped with oxygen, sulfur, or nitrogen atoms (e.g., thiophene, pyridine, piperazine, pyran, etc.) and other various functional groups.^[^ ^252^, ^253^, ^254^ ^]^ These atoms possess lone pairs of electrons, which can strongly react with uncoordinated Pb^2+^ ions. Luo et al. proposed that introducing ─NH_2_ at the ortho position can enhance the electron cloud density around the nitrogen atom of the piperazine molecule, further strengthening its electron‐donating and passivation ability to Pb^2+^ defects.^[^ ^255^ ^]^ Gao et al. reported a new modifying agent, 4‐aminotetrahydrothiopyran hydrochloride (4‐ATpHCl), whose ─S and ─NH_3_ groups passivated positively and negatively charged defects on perovskite surfaces.^[^ ^256^ ^]^ The treated film performed a higher iodine ion migration barrier from 0.63 eV to 1.19 eV, apparently reinforcing perovskite phase stability. Lv et al. utilized the multifunctional S‐ethylisothiourea hydrobromide (SEBr) to modify the surfaces of 1.67 eV and 1.77 eV perovskite films, achieving efficiency values of 22.4% and 19.9%, respectively.^[^ ^257^ ^]^ They believed that different grain surfaces harbored distinct defect types, and SEBr, with its ─S, ─NH_3_, and ═NH groups, could comprehensively passivate defects on the Pb‐I and FA‐I end‐capping surfaces to longer carrier lifespan.

Small molecules or macromolecules with a wider range of functional groups are considered capable of modifying multiple types of defects on perovskite/ETL interfaces at the same time.^[^ ^258^, ^259^ ^]^ The spatial arrangement of functional groups within the molecule framework is poised to exert a profound influence on the interaction energy with perovskites. Therefore, it underscores the critical importance of meticulous molecular design engineering. Liu et al. scrutinized the impact of a series of pyridine‐derived isomers bearing amino and carboxyl groups with different positions on 1.68 eV WBG PSCs.^[^ ^260^ ^]^ They observed that the enhanced co‐adsorption effect was obtained where two functional groups were strategically situated at the meta‐position of pyridine moiety, without compromising the interaction of the nitrogen atom in pyridine with perovskites. The optimized molecule notably decreased defect state density, inhibited ion migration channels, and achieved an efficiency of 22% with 90% retention at the MPP for nearly 1000 h. Lin et al. achieved a substantial 60‐fold increase in PLQY of perovskites with 1.6–1.8 eV bandgaps through the vapor deposition of amino‐silane molecules with secondary and primary amines.^[^ ^43^ ^]^ It was found to notably reduce the photovoltage losses of the corresponding solar cells to below 100 mV. Theoretical computations indicated the AEAPTMS exhibited the highest binding energy to surface iodine vacancies, owing to its multi‐dentate anchoring effect with the uncoordinated Pb^2+^ ions in the local and surrounding sites through Pb─N and Pb─O bonds. This strong binding force, combined with inherently hydrophobic silane moiety, rendered the devices remarkably tolerant to high temperature, high humidity, intense irradiation, and substantial current injection.

### 3.4 Buffer Layer

**Figure 7:**
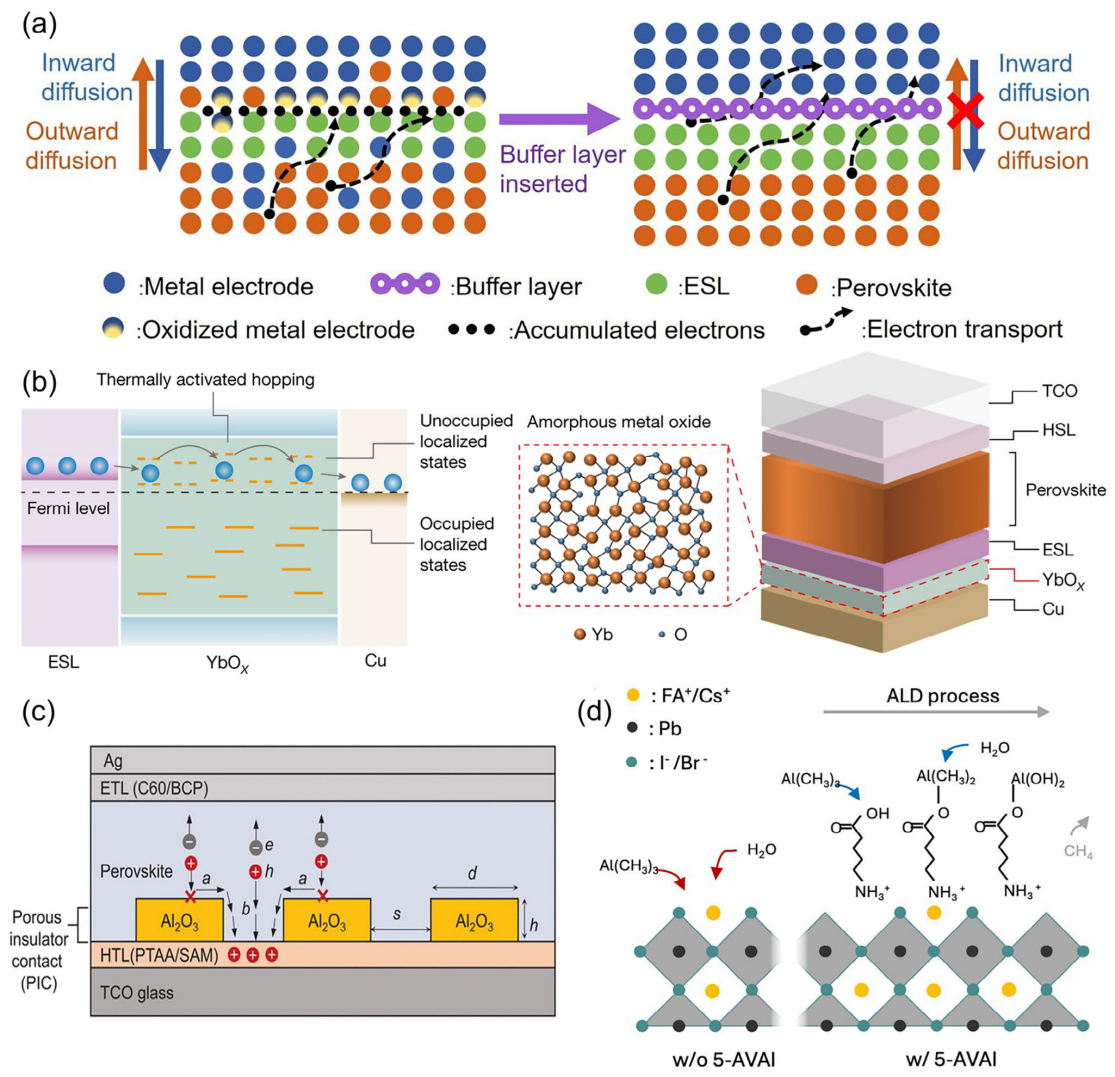
a) Schematic illustration of the functions of an ideal buffer layer between the electron‐selective layer (ESL) and the metal electrode for charge transport and blocking ion migration. b) Schematic depicts of thermally‐activated hopping carrier transport through the ESL/YbO_x_/Cu interface in a standard p‐i‐n PSC device with YbO_x_ buffer layer. a,b) Reproduced with permission.^[^ ^49^ ^]^ Copyright 2024, Springer Nature. c) Schematic diagram of carrier transport in a p‐i‐n device with a porous insulator contact structure (process a means that carriers cannot electrically tunnel through insulating materials and are forced to transport laterally, process b means that passive carriers can pass through gaps between insulating materials). Reproduced with permission.^[^ ^269^ ^]^ Copyright 2023, The American Association for the Advancement of Science. d) Schematic presenting perovskite surface without/with passivation ligands exposed to ALD precursors during AlO_x_ deposition. Reproduced with permission.^[^ ^272^ ^]^ Copyright 2024, Elsevier.

Buffer layers, known as blocking layers, commonly function with vital importance as interlayers between CTLs and electrodes. They contribute to aligning interfacial energy levels, lowering Schottky barriers, improving carrier collection efficiency, and safeguarding the inner layers against degradation by moisture and oxygen. Additionally, they are counted on to potently curb the outward migration of ions from the internal photoactive layer and the inward diffusion of ions from metal electrodes (**Figure** **7**a). This aspect is particularly critical in WBG PSCs, considering that they are more susceptible to ion migration due to photo/electrical/thermal excitations.^[^ ^30^, ^91^ ^]^ Among the various options, bathocuproine (BCP) is extensively utilized in PSCs due to its compatibility with facile solution‐processing or thermal evaporation deposition methods.^[^ ^261^ ^]^ However, BCP inherently lacks strong binding capabilities for inorganic ions. The ultra‐thin film thickness requirement in the device design makes it fail to fully cover the uneven perovskite/CTL films and leave exposed regions,^[^ ^262^ ^]^ thus ineffective in impeding ion migration. Furthermore, BCP exhibits poor heat resistance, rapidly self‐aggregating at 85 °C, which further compromises device stability.^[^ ^49^, ^263^ ^]^

Conversely, metal fluoride materials (e.g., LiF and MgF_x_) possess enhanced thermal stability, making them viable alternatives to BCP. LiF can be strategically inserted between the ETL/electrode or electrode/HTL interfaces, capitalizing on its dipole effect to lower the carrier injection barrier.^[^ ^137^, ^264^ ^]^ Their ultra‐thin nature in practical applications allows for selective charge carrier extraction through the quantum tunneling effect. More intriguingly, metal fluorides have the capability to directly modify the interfaces of perovskites and CTLs, alleviating interfacial non‐radiative recombination.^[^ ^33^, ^34^, ^59^, ^123^ ^]^ Korte accounted this for a field effect passivation induced by a combined action of a mild dipole effect and potential fixed charges.^[^ ^265^ ^]^ Whereas, Wolf's group indicated that alkali‐earth metal fluorides could isolate the perovskite/C_60_ contacts, effectively impeding the formation of resembling metal‐induced gap trap states.^[^ ^47^ ^]^ Among various alkali fluoride counterparts, they found MgF_x_‐based devices outperformed because of optimal band bending at interfaces and favorable ohmic contacts. However, subsequent investigations suggested that LiF, instead, might exhibit superior performance in terms of improving FF and reducing hysteresis, when perovskite films were pre‐treated by ammonium salts.^[^ ^59^ ^]^ The mechanisms underlying this phenomenon remain unclear and warrant further studies. Despite these advantages, over extended periods, metal fluoride materials can still negatively impact PSCs due to the migration of alkali cations and fluoride anions and their reactions with perovskites, along with the hygroscopic behavior of alkali salts.

Metal oxides are progressively becoming favored for buffer layers, owing to their versatile compositions and inherent resistance to water and oxygen. Furthermore, metal oxides provide a high degree of flexibility with aspect to film deposition techniques, involving nanoparticle‐based solution processes, magnetron sputtering, vapor‐phase deposition (ALD or CVD), and electron‐beam or thermal evaporation, catering for customization to various application requirements. Metal oxides, with their n‐type (SnO_2_, ZnO, AZO, IZO, etc.) or p‐type (MoO_3_, NiO_x_, etc.) semiconductor characteristics, can function as complementary transport layers beyond mere buffer layers. For example, recent work revealed that the work function of Ti‐doped ZnO films sputtered onto the ITO substrates could be tuned via simply Ozone UV treatment, producing a lower conduction band level than that of SnO_2_ ETL.^[^ ^266^ ^]^ Consequently, 1.66 eV‐FA_0.83_Cs_0.17_Pb(I_0.8_Br_0.2_)_3_ and all‐inorganic CsPbI_2.85_Br_0.15_ planar solar cells yielded remarkable efficiencies of 21.62% and 20.92%, respectively, which were attributed to beneficial gradient energy levels and minimized surface defects. In inverted device configurations, the outer metal oxide buffer layer can offer additional protection to inner layers from high‐energy shocks caused by sputtering transparent conductive oxide electrodes, crucial for semi‐transparent and tandem photovoltaic applications. Without such protection, otherwise, the organic transport layer would be damaged, thus rendering poor interface contact with the electrode and more severe interfacial non‐radiative recombination.

Compared to the metal oxide films deposited from nanoparticle‐based solution, ALD‐deposited films turn more compact and flatter, holding stronger capability to block the invasion of external factors, or even the post‐solvents used in the fabrication of two‐terminal TSCs. However, due to discrepancies in coefficients of thermal expansion and the roughness of an underlying perovskite film,^[^ ^267^, ^268^ ^]^ delamination would occur between ETLs and buffer layers upon heat activation. Very recently, the incorporation of a thermally evaporated ytterbium oxide (YbO_x_) buffer layer between C_60_ and Cu has been explored, exhibiting facilitated charge carrier transport property through a phonon‐assisted electron hopping mechanism by taking advantage of unoccupied in‐gap states (Figure 7b).^[^ ^49^ ^]^ This novel buffer layer was then demonstrated to significantly enhance the photovoltage and thermal stability of WBG PSCs, as evidenced by the achievement of a *V*_oc_ of 1.3 V and a thermal stability benchmark (T_80_) of 500 hours at 85 °C for a 1.77 eV FA_0.83_Cs_0.17_Pb(I_0.6_Br_0.4_)_3_ inverted device configuration, compared to BCP‐based counterparts. The observed improvements can be attributed to the effective suppression of the reaction through diffusive ions from perovskites and Cu, as well as a beneficial n‐type doping effect. Another widely employed buffer material is insulating aluminum oxides (AlO_x_) that can be incorporated into various interfaces in the form of nanoparticles (NPs) or ALD‐deposited films.^[^ ^46^, ^132^, ^269^, ^270^ ^]^ In general, AlO_x_ has the following functions: 1) the inherent bipolar fixed charge can selectively regulate the energy level alignments between interfaces, reducing the contact energy barrier; 2) increasing the electron/hole concentration ratio at the interface, mitigating the interfacial defect recombination rate; 3) physically blocking the migration of ions and volatile species (MA^+^, FA^+^, I_2_) from within the perovskite to the outside, improving device stability; 4) resistant to humidity and heat, isolating oxygen from entering. In addition, AlO_x_ porous ‘island’ regions are able to improve the wettability of substrate surfaces, beneficial for the crystallization of perovskite and the reduction of bulk defects (Figure 7c).^[^ ^269^, ^270^ ^]^ In a case of surface post‐treatment of perovskite films by ALD‐AlO_x_, trimethylaluminum (TMA), one of the precursors, was identified as a potent Lewis acid that conduced to reducing remnant Pb^0^ to Pb^2+^ and favoring the energetic alignment.^[^ ^271^ ^]^ Al^3+^ was found to penetrate the bulk phase, and passivate antisite PbX_3_^−^ defects at grain boundaries, thus impeding phase separation caused by halogen ion migration. As a result, a champion 1.66 eV WBG solar cell achieved an efficiency of 21.8% with a ≈5% increase in *V*_oc_, as well as outstanding stabilities against different aging conditions. To deposit a dense and uniform AlO_x_ layer and protect perovskite surfaces from decomposition during ALD process, Choi et al. proposed an initial surface functionalization with carboxyl‐rich 5‐ammonium valeric acid iodide (5‐AVAI) that provided more active sites for Al─O bonding and enabled deposition achieved at higher temperature.^[^ ^272^ ^]^ Thanks to the suppressed ion migration by employing this strategy, the encapsulated 1.78 eV device demonstrated a 90% efficiency retention in MPP tracking during 1‐sun (55 °C, air) aging tests. Besides, SiO_2_ NPs can also be applicable for WBG PSCs as a wettability and passivation agent.^[^ ^46^ ^]^

## 4 Applications of WBG PSCs

### 4.1 Semitransparent PSCs

Semitransparent perovskite cells embody the dual functionalities of electricity generation and light transmission. Their employment in urban buildings or agricultural greenhouses, such as facades, windows, and skylights,^[^ ^294^, ^295^, ^296^ ^]^ not only offers essential shielding and illumination but also facilitates the production of environmentally friendly and sustainable energy. This is in harmonious line with contemporary architectural paradigms that aim for sustainability and net zero carbon emission. To evaluate the performance of semitransparent photovoltaics, three key metrics demand considerations: power conversion efficiency (PCE), average visible transmittance (AVT), and light utilization efficiency (LUE). PCE, defined as the ratio of output electrical power to incident light power, mirrors the energy conversion capability of the cell system. Elevated PCE levels denote more power yield. However, it relies on a photosensitive layer with adequate thickness to capture as many photons as possible above the bandgap and an impeccably configured device that minimizes photogenerated carrier loss. Compared with low‐*E*_g_ competitors, WBG perovskites absorb the majority of in‐band incident photons in a thinner layer, owing to their high absorption coefficient and narrow absorption range, hinting at higher light transmittance. AVT, derived from the human eye's photopic response (*P(λ)*) (400–700 nm), the cell transmittance (*T(λ)*) and the light source spectrum (*S*(*λ*)), is given by:^[^ ^297^, ^298^ ^]^

(11) $$AVT=\frac{\int_{\lambda_{1}}^{\lambda_{2}}T\left(\lambda \right)S\left(\lambda \right)P\left(\lambda \right)d\lambda }{\int_{\lambda_{1}}^{\lambda_{2}}S\left(\lambda \right)P\left(\lambda \right)d\lambda }\;$$

where *λ*_1_ and *λ*_2_ represent the onset and termination wavelengths for the integration process, respectively. Halogen‐hybridized perovskite films induce a blue shift in the absorption spectra as the bandgap widens, leading to the coloration from dark reddish brown to orange or yellow, indicating an increased AVT value.^[^ ^132^, ^299^ ^]^ Meanwhile, color transformation caters to diverse architectural color aesthetics. LUE, a novel quality factor proposed by Lunt et al.,^[^ ^300^ ^]^ represents the product of PCE and AVT, which assesses the overall system efficiency. A superior LUE value is contributed by both enhanced PCE and AVT levels.

In order to attain a competitive levelized cost of electricity (LCOE) and meet the application standards for building‐integrated photovoltaics (BIPV), the LUE of devices must surpass the threshold of 2.5%, with either PCE exceeding 5% or AVT exceeding 25% at the same time.^[^ ^300^, ^301^ ^]^ Applications, such as tinted architectural glass or smart windows, have higher demands for AVT greater than 50–80%.^[^ ^300^ ^]^ For semi‐transparent solar cells with LUE > 1.5 and PCE between 2% and 5%, they can fully meet the self‐power supply of low‐power electronic devices (such as sensors, IoT devices, e‐readers, etc.), or extend the battery life of devices (such as smartwatches, tablets, smartphones, etc.) by accumulating energy over prolonged durations.^[^ ^300^ ^]^

**Figure 8:**
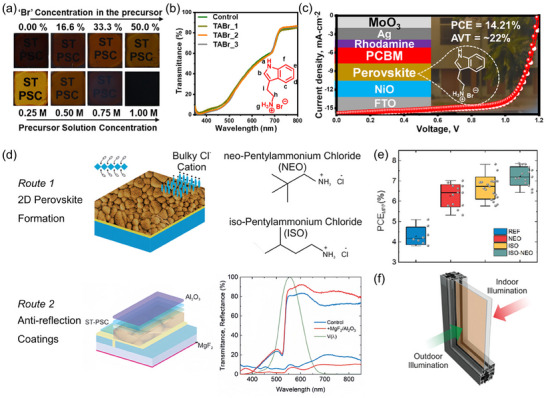
a) Digital photograph of perovskite films with different bromide content (for 0.5 m solution concentration) and varied precursor solution concentration (at 33.3% bromide concentration). b) Transmittance spectra of the perovskite films with different TABr (of which molecule structure inserted) concentration. c) *J*–*V* plot of the highest efficiency of a semi‐transparent device with a schematic device structure and a physical object. a–c) Reproduced with permission.^[^ ^298^ ^]^ Copyright 2024, American Chemical Society. d) Two technological routes to high‐efficiency semitransparent PSCs, route 1: passivation scheme using bulk chloride cations showing the chemical formula of NEO and ISO molecules, route 2: light management using anti‐reflection coating showing enhanced transmission and reduced reflection within the whole response spectra. e) Box charts graph for PCE values of multiple devices using different passivation schemes. f) Illustration of the semitransparent perovskite solar module‐powered BIPV window by bifacially absorbing outdoor/indoor illumination. d–f) Reproduced with permission.^[^ ^311^ ^]^ Copyright 2024, Wiley‐VCH GmbH.

When it comes to boosting the transparency of devices, manipulating the intrinsic perovskite layers is a straightforward approach. Transparency variation can be observed by adjusting the bromine ratio in the formulation (i.e., tuning the bandgaps) or by reducing the concentration of the precursor solution (**Figure** **8**a). However, perovskites with high bromine ratios suffer from rapid crystallization rates and poor film coverage at low concentrations. Additional interventions are expected to be imposed. For example, preparation on the mesoporous scaffold^[^ ^302^, ^303^ ^]^ or the passivated buried surface^[^ ^304^, ^305^ ^]^ can aid in fabricating smooth, continuous, and uniform films. Utilizing grid‐patterned wet deposition technology to artificially create micro‐structured light‐transmitting areas within perovskite films also contributes to the improved AVT index.^[^ ^306^, ^307^, ^308^, ^309^, ^310^ ^]^ Furthermore, replacing the opaque electrode is critical to the light transmittance of the device. Successful alternatives that have been explored involve TCOs,^[^ ^92^, ^287^, ^304^, ^311^ ^]^ metal nanowires,^[^ ^9^, ^312^ ^]^ dielectric‐metal‐dielectric (DMD) combinations,^[^ ^298^, ^313^, ^314^ ^]^ graphene,^[^ ^315^, ^316^ ^]^ and carbon nanotubes.^[^ ^317^, ^318^ ^]^ It is essential to highlight that sputtered TCOs can potentially damage the as‐prepared underlying layers, such as organic transport layers or even the perovskite layer, due to high‐energy sputtered particles, resulting in an S‐shaped JV curve. Therefore, an additional protective buffer layer is often necessary. The DMD sandwich electrode, consisting of an ultra‐thin metal film sandwiched between two high‐refractive‐index dielectric layers, leverages optical interference effects from the dielectric layers and reflections from the intermediate metal layer. This measure can maximize transparency while maintaining the high‐conductivity. Avoiding the use of transmission layers with high parasitic absorption is crucial for improving AVT as well.

Preparing high‐quality WBG perovskite films and managing energy dissipation caused by defect recombination centers are more prominent in semitransparent photovoltaic applications. The strategic interplay of reduced precursor concentrations and heightened bromine‐induced crystallization activity amplifies the likelihood of raised defect states and the formation of pinholes. Satapathi's team introduced the tryptamine hydrobromide (TABr) additive into the 1.68 eV CsFA‐based mixed halide perovskites to finely regulate crystalline quality and promote preferential (100)‐oriented crystal growth.^[^ ^298^ ^]^ Notably, varying concentrations of additives exhibited negligible influence on the films’ transmittance (Figure 8b). Instead, the ammonium moiety and aromatic amine group contained affirmatively modified negatively and positively charged defects, respectively. The resultant device achieved a PCE of 14.21%, an AVT of 22%, and a LUE of 3.12% (Figure 8c). Furthermore, the target devices demonstrated sustained humidity robustness (30–40% RH), retaining over 80% of the initial efficiency following a 1000‐h dark storage period. In another investigation, the application of 4‐chlorobutyalamine‐hydrochloride with an appropriately elongated molecular chain demonstrated pronounced efficacy in passivating perovskite/ETL interface defects, suppressing non‐radiative recombination.^[^ ^313^ ^]^ Treated devices also showcased long‐term stability against moisture and illumination. For devices based on 1.78 eV materials, a 0.14V opening voltage improvement and a 3.45% LUE were realized. In terms of the broader bandgap FAPbBr_3_ (*E*_g_ = 2.3 eV), Matteocci and his collaborators took a two‐step route, i.e., 2D/3D heterojunction passivation and light management (Figure 8d).^[^ ^311^ ^]^ Employing mixed organic salts neo‐Pentylammonium chloride (NEO) and iso‐Pentylammonium chloride (ISO) as surfactants for perovskite surface passivation led to a remarkable maximum *V*_oc_ of 1.73 V and a ≈8% PCE (Figure 8e). Later on, introducing anti‐reflection coatings of MgF_2_ and Al_2_O_3_ at separated terminals of the device substantially augmented light transmittance (Figure 8e). Ultimately, the AVT and LUE of the 1 cm^2^ device experienced increments from 64.2% and 5.06% to 70.7% and 5.73%, respectively. In addition, they also highlighted the prospects for double‐sided operation of colored transparent windows in outdoor and indoor conditions (Figure 8f). In a distinct study, the pioneering application of the same material in flexible semi‐transparent photovoltaics was demonstrated. Their satisfactory AVT and LUE performances, coupled with ISOS‐D‐1 operational stability and bending tests lasting up to 1000 h, evoked considerable enthusiasm.^[^ ^304^ ^]^ Very recently, Wu et al. proposed a crystal in situ reconstruction strategy by anchoring acid‐anion‐dominated agents to initiate compression stress and stabilize phase homogeneity against solar irradiance.^[^ ^314^ ^]^ CsPbIBr_2_‐based semitransparent cells set a record of 5.8% LUE with 41.58% AVT and 13.97% PCE.

### 4.2 Tandem PSCs

The design of TSCs, which takes advantage of the minimal thermalized carrier losses in WBG semiconductors and the broad absorption spectra in NBG semiconductors, has been demonstrated to surpass the S‐Q limit of single‐junction solar cells.^[^ ^7^, ^23^ ^]^ Taking the example of a two‐junction TSC, two stack configurations are quite mainstream.^[^ ^20^ ^]^ For a four‐terminal (4T) configuration, two sub‐cells are either mechanically stacked or coupled with a spectral beam splitter. This setup has low fabrication requirements for each sub‐cell which separately achieves its maximum PCE through optical coupling. However, it demands additional precise circuitry wiring, multiple substrates, and a complicated assembly process, leading to high manufacturing costs. Meanwhile, parasitic absorption and reflection in TCO/Glass substrates and TCO top electrodes result in considerable optical loss. In contrast, a two‐terminal (2T) configuration involving a single interconnecting layer in which the opposite charge carriers from the two sub‐cells recombine offers simplicity in circuit design. Despite its distinct strengths in lightweight and cost‐efficiency, greater demands are imposed on the electrical property (Ohmic contacts), optical coupling (high transmittance for long‐wavelength spectra), and spatial solvent barrier property (protection of underlying layers from being damaged by the upper layer preparation). Moreover, in the case of 2T design, considerations must be taken into the current matching between sub‐cells. Theoretically, although 2T and 4T TSCs show similar maximum limiting efficiencies (≈46%), 4T holds a broader selection of material bandgaps.^[^ ^7^ ^]^

**Table** **2** presents the photovoltaic performance of some of the most advanced TSCs currently available. As far as bandgap is concerned, cutting‐edge TSCs have all gravitated towards bandgap pairings that align theoretically. For silicon rear cells with a 1.12 eV bandgap, the optimal options lie in WBG perovskites with bandgaps spanning from 1.67 to 1.75 eV. WBG materials with ≈1.8 eV bandgaps are ideal for the lead‐tin mixed perovskites possessing larger bandgaps (≈1.25 eV). While, for the NBG organic materials listed in the table, which may have bandgaps around ≈1.35 eV, 1.8 or 1.9 eV WBG perovskites are commonly selected. This hints at the notion that previous theoretical contemplations have effectively guided practical applications. Looking at the developmental landscape, Si‐based TSCs exhibit top‐tier performance, with multiple reported breakthroughs surpassing 30% PCE. Following closely are the all‐perovskite TSCs, with the majority of reported efficiencies outperforming those of the best single‐junction PSCs, and steadily advancing towards the 30% PCE frontier. Efforts directed towards perovskite/organic TSCs are ongoing, potentially hindered by the efficiency limitations of sole organic solar cells. Certain research has concentrated on the development of three‐junction monolithic TSCs, boasting *V*_oc_ over 3 V. These efficiency advances are primarily driven by the reduction in open‐voltage deficits of WBG PSCs through defect optimizations across different component layers, thus alleviating non‐radiative recombination.^[^ ^26^, ^44^, ^46^, ^137^, ^158^, ^209^ ^]^ Concurrently, defect management improves the stability of devices tremendously.^[^ ^35^, ^36^, ^59^, ^319^ ^]^

**Table 2:** Summary of photovoltaic performance of state‐of‐the‐art multi‐junction PSCs.

*E*_g_ [eV] of WBG sub‐cell	Type of bottom sub‐cell(s)	*V*_oc_ [V]	*J*_sc_ [mA cm^−2^]	FF [%]	PCE [%]	Refs.
4T stacked tandem solar cells						
1.77	Si	–	–	–	28.09	[183]
1.66	Si	–	–	–	27.16	[273]
1.67	Si	–	–	–	33.10	[189]
1.77	Pb–Sn mixed perovskite	–	–	–	26.09	[183]
1.77	Pb–Sn mixed perovskite	–	–	–	27.28	[241]
1.68	Pb–Sn mixed perovskite	–	–	–	26.72	[185]
1.77	Pb–Sn mixed perovskite	–	–	–	26.9	[206]
1.68	Pb–Sn mixed perovskite	–	–	–	28.07	[242]
1.67	Pb–Sn mixed perovskite	–	–	–	28.08	[163]
1.77	Pb–Sn mixed perovskite	–	–	–	27.38	[256]
1.68	Pb–Sn mixed perovskite	–	–	–	28.07	[242]
1.67	Pb–Sn mixed perovskite	–	–	–	28.35	[276]
1.75	Pb–Sn mixed perovskite	–	–	–	28.08	[287]
1.77	Pb–Sn mixed perovskite	–	–	–	28.46	[158]
1.77	Organic	–	–	–	25.28	[183]
2T monolithic tandem solar cells						
1.67	Si	1.764	19.99	80.15	28.24	[54]
1.67	Si	1.929	19.58	81.54	30.8	[319]
1.65	Si	1.9	19.59	82.5	30.7	[271]
1.66	Si	1.819	20.64	75.92	28.5	[46]
1.67	Si	1.91	19.1	79.1	28.9	[209]
1.71	Si	1.902	17.1	75.11	24.29	[92]
1.68	Si	1.96	20.01	78.48	30.78	[279]
1.71	Si	2.024	17.02	79.13	27.27	[161]
1.65	Si	1.9	20.12	79.8	30.5	[63]
1.69	Si	1.966	20.76	83	33.89	[44]
1.67	Si	1.873	20.42	80.1	30.64	[275]
1.68	Si	1.95	19.76	79.95	30.78	[159]
1.68	Si	1.926	20.71	79.39	31.67	[138]
1.68	Si	1.931	19.89	81.54	31.32	[165]
1.68	Si	1.903	19.08	79.57	28.89	[281]
1.68	Si	1.87	20.65	83.34	32.13	[26]
1.73	Pb–Sn mixed perovskite	2.109	15.38	82.5	26.76	[185]
1.77	Pb–Sn mixed perovskite	2.11	15.37	83.13	27.01	[207]
1.73	Pb–Sn mixed perovskite	2.115	15.29	82.5	26.68	[31]
1.79	Pb–Sn mixed perovskite	2.12	15.43	79.72	26.08	[326]
1.77	Pb–Sn mixed perovskite	2.13	15.52	82.36	27.22	[327]
1.77	Pb–Sn mixed perovskite	2.14	15.69	82.83	27.81	[215]
1.77	Pb–Sn mixed perovskite	2.12	16.02	83.88	28.49	[328]
1.79	Pb–Sn mixed perovskite	2.21	15.1	81.7	27.3	[329]
1.77	Pb–Sn mixed perovskite	2.17	16.4	80.2	28.5	[45]
1.78	Pb–Sn mixed perovskite	2.11	15.8	81	27	[50]
1.79	Pb–Sn mixed perovskite	2.113	15.85	81.4	27.27	[38]
1.8	Pb–Sn mixed perovskite	2.15	15.5	80.2	26.8	[330]
1.8	Pb–Sn mixed perovskite	2.045	15.98	79.9	26.1	[238]
1.77	Pb–Sn mixed perovskite	2.12	16.01	80.36	27.23	[250]
1.77	Pb–Sn mixed perovskite	2.14	15.98	79.3	27.1	[257]
1.77	Pb–Sn mixed perovskite	2.13	15.59	82.73	27.47	[255]
1.77	Pb–Sn mixed perovskite	2.18	15.57	84.8	28.42	[331]
1.77	Pb–Sn mixed perovskite	2.10	16.58	79.91	27.76	[167]
1.77	Pb–Sn mixed perovskite	2.12	15.49	82.43	27.11	[164]
1.77	Pb–Sn mixed perovskite	2.131	16.21	82.6	28.28	[166]
1.79	Pb–Sn mixed perovskite	2.11	15.63	83.64	27.66	[290]
1.79	Pb–Sn mixed perovskite	2.11	16.49	79.81	27.70	[208]
1.79	Organic	2.15	14.68	81.03	25.54	[121]
1.91	Organic	1.84	14.34	73.76	19.42	[292]
1.83	Organic	2.12	14.68	82.97	25.82	[36]
1.83	Organic	2.135	14.66	82.82	25.92	[332]
1.81	Organic	2.151	14.36	81.65	25.22	[39]
1.91	Organic	2.152	13.89	80.57	24.07	[199]
Triple‐junction monolithic tandem solar cells						
1.93	1.55 eV perovskite + Si	3.132	11.58	76.15	27.62	[35]
1.97	1.61 eV perovskite + Pb–Sn mixed perovskite	3.33	9.7	78	25.1	[293]
1.96	1.53 eV perovskite + Si	2.995	11.76	71	25	[137]

**Figure 9:**
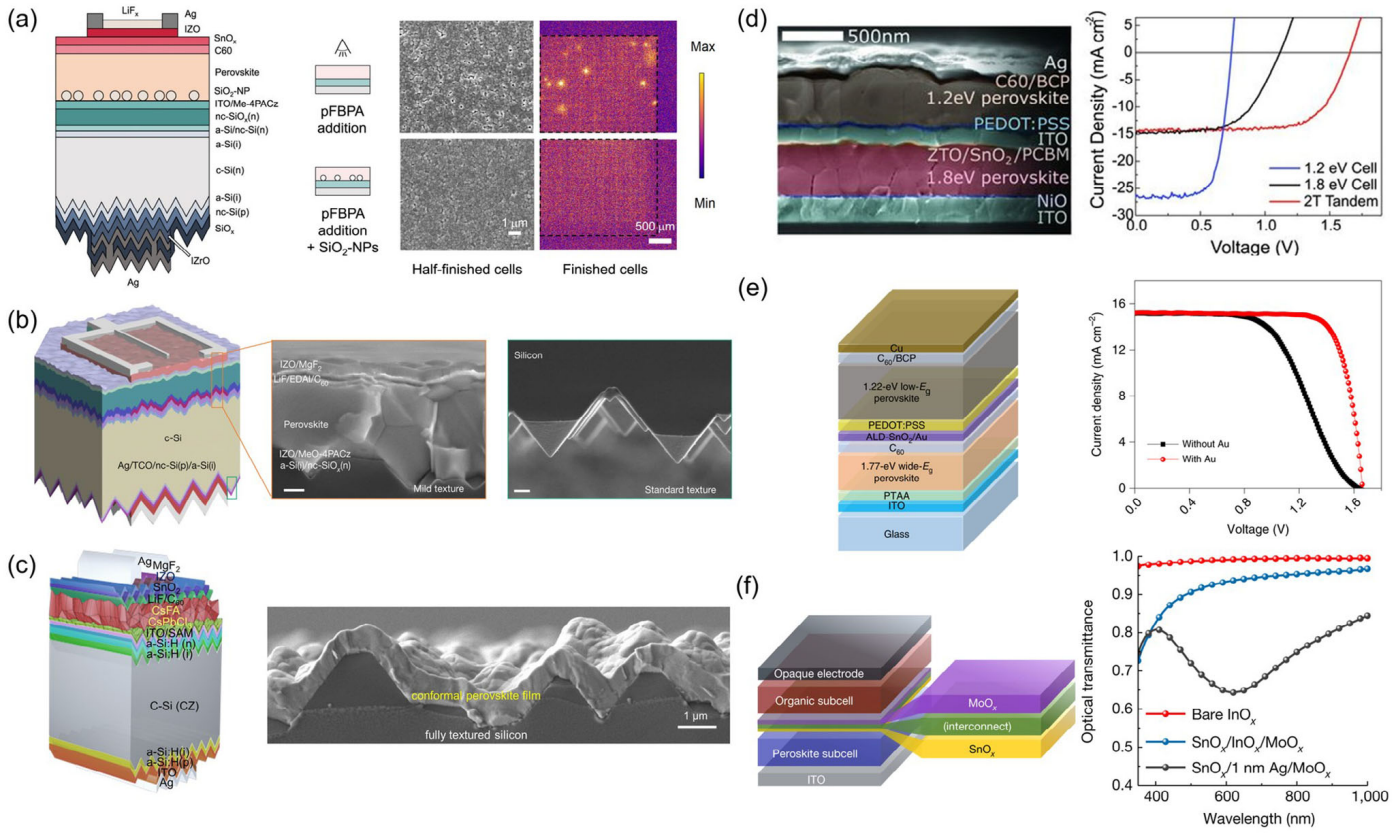
a) Structural diagram of a back‐textured Si/perovskite TSC, and SEM and lock‐in thermography images of half‐finished perovskite films and fully‐finished devices without/with SiO_2_ NPs treatment. Reproduced under the terms of the CC‐BY Creative Commons Attribution 4.0 International License (https://creativecommons.org/licenses/by/4.0/).^[^ ^48^ ^]^ Copyright 2024, The Authors, published by Elsevier. b) Structural diagram of an asymmetric double‐side‐textured Si/perovskite TSC, selectively showing the SEM cross‐section images of the perovskite deposited on mildly textured front surface and the standard‐sized pyramid texture on the rear side. Reproduced with permission.^[^ ^44^ ^]^ Copyright 2024, Springer Nature. c) Structural diagram a SEM cross‐section image of a fully‐textured Si/perovskite TSC. Reproduced with permission.^[^ ^26^ ^]^ Copyright 2024, Elsevier. d) a SEM cross‐section image of the 2T all‐perovskite tandem device in which sputtered ITO as the interconnection layer, alongside the corresponding photovoltaic characteristics. Reproduced with permission.^[^ ^320^ ^]^ Copyright 2016, The American Association for the Advancement of Science. e) Device structure of an all‐perovskite TSC where ALD‐SnO_x_/Au serves as tunnel recombination layer, with the *J*–*V* curves of devices without and with Au shown beside. Reproduced with permission.^[^ ^321^ ^]^ Copyright 2019, Springer Nature. f) Schematic of a perovskite/organic TSC with InO_x_/ or Ag/MoO_x_ as interconnect, with comparative optical transmittance displayed on the right. Reproduced with permission.^[^ ^322^ ^]^ Copyright 2022, Springer Nature.

The overall efficiency of TSCs critically hinges on specific strategies. To minimize light loss, silicon‐based TSCs usually utilize textured crystalline silicon wafers to enhance the optical path in the active region and reduce reflectivity in the interlayer. These substrates can feature micro/nano‐scale pyramid/cone patterns on the back or both sides. The backside textured silicon substrates have a smooth front surface, akin to ITO glass substrates, facilitating the deposition of perovskite on this side. To improve the surface wettability of ITO/Me‐4PACz, Turkay et al. coated a layer of SiO_2_ nanoparticles. The uniform, dense, and high‐quality perovskite films were confirmed by SEM and bias‐thermal imaging techniques (**Figure** **9**a).^[^ ^48^ ^]^ Independently certified TSC efficiencies reached up to 30.9%. They also emphasized the importance of backside protection for silicon cells and proposed the use of an Ag/PECVD‐SiO_x_/Ag stack to effectively prevent mechanical damage. Double‐sided texturing has a dual reflection effect, capturing infrared light better than the single‐sided, reducing primary reflections. Liu et al. used asymmetrically sized double‐sided textured silicon sub‐cells, aiming to ensure full coverage of the perovskite film over a mild‐sized pyramid‐textured front for improved *V*_oc_ and FF (Figure 9b).^[^ ^44^ ^]^ The highly‐textured backside was retained to keep its optical and electrical performance advantages. Fully textured TSCs suggest that perovskite top cells are prepared along the pyramidal texture, favoring the consistency of photoactive layer thickness and carrier transport paths. Liu et al. reported a vacuum‐deposited CsPbCl_3_ buried buffer layer to construct vertical 3D/3D strained heterostructures (Figure 9c).^[^ ^26^ ^]^ Combined with a fully‐textured light management strategy, the TSC exhibited a short‐circuit current density of 20.65 mA cm^−2^. Lately, Kan et al. screened copper(I) thiocyanate for co‐deposition with perovskite ink, which turned out to form benign hole‐collecting contacts and passivate grain boundaries at buried interface.^[^ ^138^ ^]^ This strategy effectively solved the issues of interfacial defects, non‐conformal deposition, and de‐wetting of perovskite on ITO/silicon substrates. The champion tandem device delivered a certified efficiency of 31.46% and performed excellent operational stability of more than 1000 h under damp‐heating conditions.

For solution‐prepared perovskite/perovskite and perovskite/organic solar cells, the quality of interconnect layers is paramount to final performance. Initially, sputter‐deposited ITO with a typical thickness over 100 nm, sufficient to protect the underlying films from solvents, was frequently used (Figure 9d).^[^ ^320^ ^]^ An additional rigid inorganic or thick organic layer prior to sputtering could protect against bombardment by high‐energy particles, ensuring the output of a regular TSC testing curve. However, the parasitic absorption drawback of ITO is evident, which impacts device photocurrents. Subsequently, a combination of the thin but dense ALD‐deposited SnO_x_ (≈25 nm) layer combined with the ultrathin metal (≈1 nm) film has proven effective as an alternative (Figure 9e). This approach not only set the stage for the fabrication of recombination layers for all‐perovskite TSCs, but also inspired the development of metal/MoO_x_ combinations in perovskite/OSC community. Metal nanoclusters are essential because they overcome the problem of impaired carrier extraction or mobility brought about by the direct contact of different n‐/p‐type semiconductors.^[^ ^323^ ^]^ Yet, the high reflectance of metals diminishes the transmittance of long‐wavelength light, thus affecting the current response of rear cells. Brinkmann et al. proposed the use of ALD‐deposited InO_x_ with metallic nature as a substitute for metal clusters in their perovskite/OSC devices, which significantly reduced optical losses (Figure 9f).^[^ ^322^ ^]^ This work guides our research on strong opto‐electrically coupled interconnect layers.

In addition, some works have finely optimized the quality of the interfacial layer. For example, Zhu et al. introduced additional thin layers of PEDOT:PSS to solve the problem of sparse and localized aggregation of SAM molecules directly deposited on the interconnect layer. Comparable approaches, like ITO nanocrystals or pre‐sputtered NiO_x_ layers, have also proved successful.^[^ ^324^, ^325^ ^]^ In addition, Liu et al. pointed out that the aggregation of C_60_ affected the subsequent chemisorption and film‐forming quality of ALD‐SnO_x_, resulting in extra non‐radiative recombination.^[^ ^54^ ^]^ Tan's team achieved uniform contact of C_60_ by introducing a custom two‐dimensional modifying layer on the top perovskite/ETL interface.^[^ ^45^ ^]^ Their all‐perovskite TSCs delivered a high efficiency of 28.5%.

### 4.3 Indoor photovoltaics

**Figure 10:**
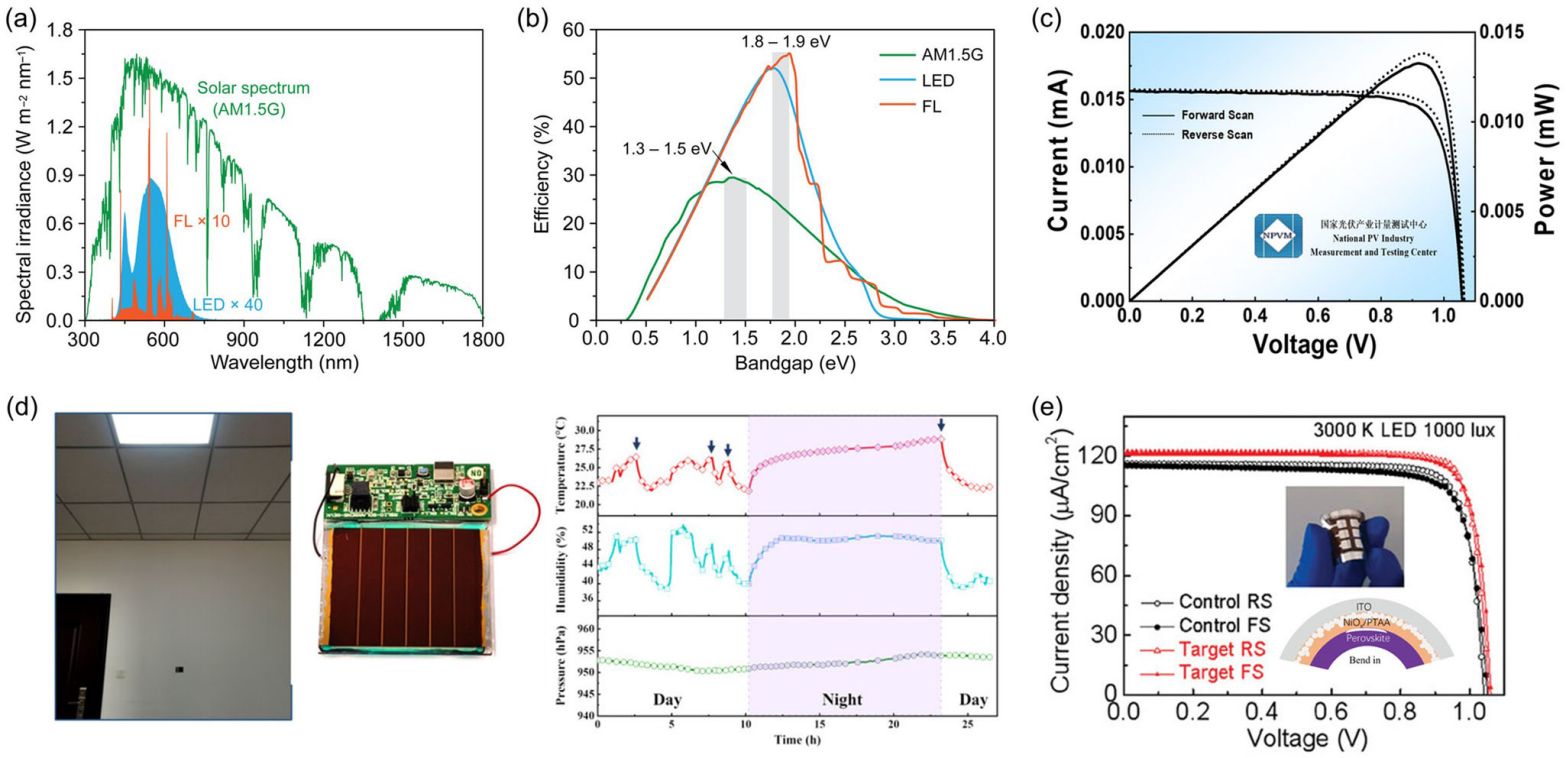
a) Characteristic spectral of AM1.5G solar, 2700 K LED, and 1000 lux FL. b) Bandgap‐dependent S‐Q limits under illumination from one sun, 1000 lux LED, and 1000 lux FL. a,b) Reproduced with permission.^[^ ^336^ ^]^ Copyright 2022, The American Association for the Advancement of Science. c) A certificated indoor current‐voltage characteristic with an efficiency of 44.72%. Reproduced with permission.^[^ ^338^ ^]^ Copyright 2024, The Royal Society of Chemistry. d) Photograph of a perovskite module connected to an IoT evaluation board in an office setting, showcasing temperature, humidity, and pressure data transmission during LED illumination. Reproduced with permission.^[^ ^339^ ^]^ Copyright 2024, Wiley‐VCH GmbH. e) *J*–*V* curves of the control and target flexible perovskite photovoltaic cells under 1000 lux LED illumination, with a photograph of a physical bending device and a diagram of a bending device inserted therein. Reproduced with permission.^[^ ^340^ ^]^ Copyright 2024, Wiley‐VCH GmbH.

The rapid evolution of IoT has catalyzed the exponential growth of node electronic devices.^[^ ^11^, ^333^, ^334^ ^]^ Conventional battery‐powered routes are becoming inadequate to fulfill the application demands. Considering that a certain portion of these devices are deployed in indoor spaces, using photovoltaic cells to convert ambient artificial light energy into usable electricity emerges as a highly promising solution. Although the indoor light intensity catering for human activities only ranges 200–1000 lux, corresponding to a power density of 50–300 µW cm^−2^, two–three orders of magnitude lower than that of sunlight, IPVs are evaluated to have sufficient capacity to power wireless communication protocols and personal and household devices consuming power in the range of several to hundreds of microwatts.^[^ ^4^ ^]^ In IPV application field, WBG perovskite materials are widely acknowledged as prime candidates. For one thing, in contrast to the broad solar spectrum encompassing ultraviolet to infra‐red wavelengths, the emission spectrum of artificial light sources (e.g. FL, WLED, U30, etc.) solely falls within the visually visible range of 400–700 nm (**Figure** **10**a). This spectral composition aligns perfectly with the absorption characteristics of WBG perovskites, resulting in minimized thermal loss of carriers. By extending S‐Q theory to the IPV domain, the optimal bandgap is theoretically estimated to be 1.8–2.0 eV with the maximum conversion efficiency reaching over 50–60% (Figure 10b).^[^ ^11^, ^335^, ^336^ ^]^ Noted that such range variation is intricately linked to various spectral shapes influenced by the types or color temperatures of light sources.^[^ ^336^ ^]^ For another thing, photo‐induced phase segregation of bromide‐rich perovskites can be overcome under low‐intensity indoor lighting conditions, offering advantages for further exploration based on halogen hybrid WBG perovskites.^[^ ^337^ ^]^

However, trap‐assisted recombination losses in bulks and at interfaces of WBG devices turns to become the dominant player affecting IPV performance. According to the dependence of photovoltage on light intensity ($V_{oc}=\frac{nkT}{q}\;\ln\left(\frac{I}{I_{0}}+1\right)$),^[^ ^101^ ^]^ a *V*_oc_ of 1.20 V obtained under 1‐sun illumination ideally (ideality factor n = 1) translates to a value of 1.05 V at 1000 lux lighting. When *n* is greater than unity, SRH recombination inhibits the device from approaching this indoor photovoltage. Given that the high efficiency of WBG PSCs hinges on high *V*_oc_, efforts directed at passivating intrinsic defects in photoactive materials and interfacial defects across the entire device remain a top priority. **Table** **3** presents the performance metrics of recently reported high‐efficiency WBG perovskite IPVs, alongside the strategies employed to improve their capabilities.

**Table 3:** Summary of indoor photovoltaic performance of state‐of‐the‐art IPVs.

Strategies	*E*_g_ [eV]	Illumination	Light Intensity [lux]	i*V*_oc_ [V]	i*J*_sc_ [µA cm^−2^]	iFF [%]	iPCE [%]	Refs.
Additive engineering								
OAmI:CHCl_3_	1.71	U30	1000	1.069	171.91	82.3	44.72	[338]
ANT	1.9	3200K LED	1000	0.965	158	73.5	29.47	[343]
HABr‐ThEABr	1.79	3800K LED	1000	1.11	154.8	76.63	41.58	[339]
Interface passivation								
THB	1.71	LED	1000	1.053	123.6	81.37	34.15	[259]
PP3	1.72	LED	1000	0.98	147	84.3	40.1	[341]
KCl	2.3	LED	1200	1.16	93	68.2	18.7	[304]
BA_2_PbI_4_	1.7	LED	200	1.01	34.41	80.81	43.7	[344]
TMABF4	1.72	3376K LED	984	–	–	–	38.98	[345]
	1.78	3376K LED	984	–	–	–	39.23	
Charge transport layer optimization								
PCBM:CMC	1.72	3000K LED	175	1.05	26.4	79.86	29.62	[346]
NiO_x_/PTAA:1wt% F4‐TCNQ	1.74	3000K LED	1000	1.06	121.5	84.57	36.11	[340]
NiO_x_/MeO‐2PACz	1.65	3000K LED	1000	1.04	288.45	79.8	41.39	[342]
NiO_x_/4PACDB	1.65	3000K LED	1000	1.04	293.14	77.3	42.05	[342]

Ma et al. ascertained that trichloromethane (CHCl_3_) enhanced the crystallinity of perovskite, while oleylammonium iodide (OAmI) fostered the formation of 2D/3D heterostructures at grain boundaries.^[^ ^338^ ^]^ By the dual additive approach, 1.71 eV‐WBG IPV delivered a certified indoor PCE (IPCE) of 44.71% under U30 illumination at 338 µW cm^−2^ (Figure 10c), attributed to significantly reduced bulk trap defects. Similarly, Li et al. incorporated hexaneammonium organic cations (HA^+^) to create 2D perovskites, coupled with capping the uppermost surface with 2‐thiopheneethylammonium (ThEA^+^) ions.^[^ ^339^ ^]^ 1 cm^2^ device achieved a 31.33% IPCE with a maximum output power of ≈120 µW cm^−2^ at 1200 lux LED intensity. More excitingly, a successful 24‐h monitoring was conducted by capturing environmental data (humidity, temperature, and gas pressure) from an IPV‐driven IoT integrated circuit in an office environment via the mobile client (Figure 10d). Moreover, Hu's team leveraged tris[2‐(diphenylphosphino)ethyl]phosphine (PP3) ligands featuring well‐balanced electronic and steric properties to modulate the perovskite/P3HT interface, thus passivating deep‐level anti‐site defects and enhancing structural stability.^[^ ^339^, ^341^ ^]^ Shi et al. found bilayers of NiO_x_ and SAM molecules played a positive role in regulating perovskite growth, promoting carrier transport, and suppressing interface defect generation. These representative strategies in interface passivation engineering and CTL optimization have propelled IPV devices to achieve performance levels exceeding 40%. Additionally, flexible IPV devices have great potential in portable and wearable electronic applications. Zhang et al. introduced mesoporous NiO_x_ nanoparticles into the buried interface to augment the mechanical engagement between perovskites and substrates.^[^ ^342^ ^]^ This method not only produced record efficiencies of 36.11% for small‐area devices (Figure 10e) and 31.69% for large‐area modules, respectively, but maintained 80% efficiency after being bent 2000 times.

A critical point deserving special attention is that the current high‐efficiency WBG IPVs predominantly rely on materials that deviate from the optimal bandgaps specified in IPV theory. The larger the bandgap, the more serious the interface non‐radiative recombination suffered.^[^ ^108^ ^]^ Therefore, the IPV performance of materials with ideal bandgaps is still far behind. Furthermore, the indoor photovoltage reported in most cases is still hovering around 1.08 V, signaling ample room for improvement.

## 5 Conclusion and Outlook

The high‐efficiency performance and stable operation of WBG PSCs are fundamental for their prospective applications. In this review, we first explore in detail the mechanism of how the intrinsic defect ecosystem within devices influences photostability and photovoltage loss. We identify deep‐level defects (such as Pb^2+^, I^−^, PbX_3_^−^, Pb^0^, PbX_2_, etc.) as primary targets to overcome. These defects induce light‐induced secondary reactions and non‐radiative recombination of internal charge carriers to a significant extent. Conversely, although shallow energy‐level defects may not directly impact voltage output, they, in turn, indirectly degrade devices by decreasing ion migration activation energy, aggravating lattice strain due to light‐induced charge accumulation at defective sites, and triggering phase segregation. The energy level alignment and inherent defect states of CTLs also require attention. The former can lead to an exponential increase in trap‐assisted recombination at interfaces, while the latter may present a bottleneck for photovoltage saturation. Taken together, to achieve high voltage and long‐term stability, defect management should be holistic, involving all device components and their interfaces.

In line with this principle, this review systematically summarizes the recent strategies reported for high‐efficiency performance. Manipulations in compositions, additives, solvents, fabrication techniques, and buried interface modifications play integral roles in refining the crystalline quality of perovskite layers, homogenizing halogens, releasing strain, inhibiting ion migration, mitigating phase separation, and impeding defect state formation. In particular, we appeal to the modulation of the crystalline orientation of WBG PSCs by phosphate‐based compounds or organic modifiers with strongly electronegative atoms or groups. (111)‐oriented mixed halide perovskites offer superior natural stability, making it a preferred option. Additionally, there is a pressing need to investigate the influence of the dipole moments of these chemicals on bandgap tuning. Post‐treatment surface passivation not only improves defect‐rich surfaces but also forms a favorable energetic transition layer in situ at interfaces. Amino‐functionalized organic compounds play an important role in this regard, warranting deeper exploration into their design, and mechanism interpretations in defect passivation and charge transport properties. The selection of SAM charge molecules suitable for WBG perovskites remains limited, especially for those with wider bandgaps and lower valence band levels. The design of SAM molecules should prioritize terminal‐function groups modified by asymmetric moieties to ensure a more homogeneous and comprehensive coverage. Also, their charge carrier mobility would benefit from the use of conjugated aromatic spacers. Blocking layer not only obstruct the pathway of mobile ions but also shield against external moisture and oxygen. More importantly, they also exhibit potential for enhancing device performance when closely integrated with perovskite layers. This is a direction that deserves to be thoroughly explored and researched.

It is evident that the future design of efficient WBG PSCs will not be a singular optimization improvement but a collective advancement of all components. Therefore, future research directions should build upon established effective strategies and focus on the compatibility between multiple strategies while strengthening analysis and mechanism explanations. From the application standpoint, the application scope and potential of WBG PSCs are commendable. Semitransparent cells achieve high AVT by sacrificing the thickness of the light‐absorbing layer, which imposes stricter requirements for device quality, especially in reducing defect states. The photo‐induced instability is no longer a major concern with regard to indoor applications; instead, trap‐assisted non‐radiative recombination losses will determine the ultimate indoor photovoltaic performance. Hence, there is a need to enhance the suppression and reduction of deep‐level defects. TSCs are widely embraced in commercial and research fields due to their significant potential to decrease LCOE, with ample room for further development. Emphasizing light management and reducing parasitic absorption in non‐active layers will be one effective way to enhance photocurrent and efficiency.

## Conflict of Interest

The authors declare no conflict of interest.
